# Comparative Genomics of the *Aeromonadaceae* Core Oligosaccharide Biosynthetic Regions

**DOI:** 10.3390/ijms18030519

**Published:** 2017-02-28

**Authors:** Gabriel Forn-Cuní, Susana Merino, Juan M. Tomás

**Affiliations:** Department of Genética, Microbiología y Estadística, Universidad de Barcelona, Diagonal 643, 08071 Barcelona, Spain; gabrifc@gmail.com (G.-F.C.); smerino@ub.edu (S.M.)

**Keywords:** *Aeromonas*, genomics, inner core oligosaccharide, outer core oligosaccharide, lipopolysaccharide

## Abstract

Lipopolysaccharides (LPSs) are an integral part of the Gram-negative outer membrane, playing important organizational and structural roles and taking part in the bacterial infection process. In *Aeromonas hydrophila*, *piscicola*, and *salmonicida*, three different genomic regions taking part in the LPS core oligosaccharide (Core-OS) assembly have been identified, although the characterization of these clusters in most aeromonad species is still lacking. Here, we analyse the conservation of these LPS biosynthesis gene clusters in the all the 170 currently public *Aeromonas* genomes, including 30 different species, and characterise the structure of a putative common inner Core-OS in the *Aeromonadaceae* family. We describe three new genomic organizations for the inner Core-OS genomic regions, which were more evolutionary conserved than the outer Core-OS regions, which presented remarkable variability. We report how the degree of conservation of the genes from the inner and outer Core-OS may be indicative of the taxonomic relationship between *Aeromonas* species.

## 1. Introduction

Aeromonads are an heterogeneous group of Gram-negative bacteria emerging as important pathogens of both gastrointestinal and extraintestinal diseases in a great evolutionary range of animals: from fish to mammals, including humans [1]. In recent years, as our knowledge about *Aeromonas* taxonomy, biology and pathogenicity has increased, and the number of reported infections caused by these microorganisms in healthy and immunocompromised patients has also spiked [2]. Despite the fact that, in humans, the most common complications derived from these pathogens are mild and easily tractable, they can present a serious risk in immunocompromised patients, causing severe septicaemia and even death [3]. Therefore, increasing our knowledge on the virulence factors governing aeromonad pathogenicity is of crucial importance to prevent the increasing complications caused by these bacteria.

**Figure 1:**
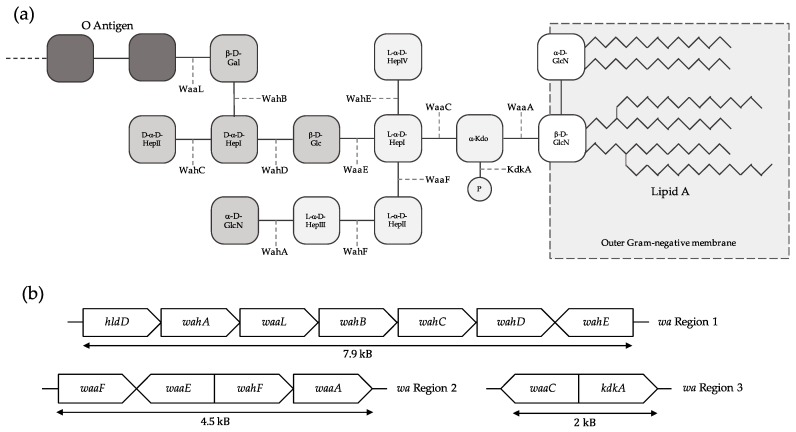
Structure and genomic organization of the *Aeromonas piscicola* AH-3 lipopolysaccharide (LPS). (**a**) Chemical structure of the *A*. *piscicola* AH-3 LPS. From clearer to darker, the carbohydrates of the inner core oligosaccharide, outer core oligosaccharide, and O-antigen (only the first two molecules) are shown. The name of the proteins catalysing each reaction is shown in each link between components; and (**b**) Genomic organization of the *A*. *piscicola* AH-3 genes coding for the proteins taking part in the LPS biosynthesis.

The virulence and pathogenicity of *Aeromonas* is multifactorial, varies between species and strains, and has been linked to, among others, toxins, flagella, secretion systems, outer-membrane proteins and capsules, and surface polysaccharides, such as lipopolysaccharides (LPSs) [4]. LPSs, also known as endotoxins, are an integral part of the outer membrane for the great majority of Gram-negative bacteria, covering approximately the 75% of its surface and playing a crucial role on its organization and structure [5]. Although the exposed sections of LPSs are highly variable between species—and sometimes even between strains—LPSs molecules follow the same structural architecture depicted in Figure 1: a hydrophobic lipid component, the lipid A, bound to a hydrophilic polysaccharide [5]. The polysaccharide is composed by the core oligosaccharide (Core-OS), and the more variable O-specific chain (O-antigen), which may be present (Smooth LPS) or not (Rough LPS). The Core-OS can be further subdivided into the inner core and outer core. On the one side, the inner core, containing a high proportion of unusual sugars, predominantly one to three 3-deoxy-d-manno-oct-2-ulosonic acid (Kdo) residues and two or three heptoses (Hep), tends to be evolutionarily conserved within and a taxonomic family or genus, and is the part bound to the lipid A [6]. On the other side, the outer core is usually formed by common sugars—as hexoses and hexosamines—present more variability, and this is the region bound to the O-antigen, if present [6].

While the *Aeromonas* LPS follows the same architectural pattern as the rest of Gram-negative bacteria, this molecule displays remarkable diversity across species of this genus. We previously reported the molecular structure of the LPS from wild-type *A*. *hydrophila* AH-1 [7], *A*. *piscicola* AH-3 (formerly *A*. *hydrophila* AH-3) [8] and *A*. *salmonicida subsp*. *salmonicida* [9]. Furthermore, we characterized the genes coding for the proteins taking part in the assembly of both the inner and outer LPS core of these species, which are located in three different genomic regions: the *wa* Regions 1 to 3 [8,9]. Roughly, *wa* Region 1 contains genes related to the outer core, whereas *wa* Regions 2 and 3 code for enzymes related to the inner core biosynthesis (Figure 1). To date, the conservation and characterization of these LPS biosynthetic regions, which may be a key factor governing pathogenicity, remains to be studied in the rest of *Aeromonadaceae*. In this article, we analyse the synteny of the three Core-OS biosynthesis gene clusters across all the publicly available *Aeromonas* genomes and predict, for the first time, a conserved inner LPS oligosaccharide core substructure in all the species of *Aeromonas* genus.

## 2. Results

A total of 170 genomes comprising a variable number of strains and biovars from the Aeromonas species *A. allosaccharophila*, *A. aquatica*, *A. australiensis*, *A. bestiarum*, *A. bivalvium*, *A. caviae*, *A. dhakensis*, *A. diversa*, *A. encheleia*, *A. enteropelogenes*, *A. eucreophila*, *A. finlandiensis*, *A. fluvialis*, *A. hydrophila*, *A. jandaei*, *A. lacus*, *A. media*, *A. molluscorum*, *A. piscicola*, *A. popoffii*, *A. rivuli*, *A. salmonicida*, *A. sanarellii*, *A. schubertii*, *A. simiae*, *A. sobria*, *A. taiwanensis*, *A. tecta*, *A. veronii*, and other uncharacterized strains (*Aeromonas sp.*) were retrieved from the National Center for Biotechnology Information genome database (Available online: https://www.ncbi.nlm.nih.gov/genome/). The complete list of genomes and strains used in this study is available at the Table S1. Using the sequence of the *A. hydrophila* ATCC7966 TetR, WaaA, and WaaC proteins, we located the *wa* Regions 1, 2, and 3, respectively, in each of the genome assemblies, thus confirming the conservation of these LPS biosynthetic regions inside *Aeromonas*.

### 2.1. Synteny of the wa Regions 2 and 3

The genomic *wa* Regions 2 and 3 were found in all the *Aeromonadaceae* genomes studied, highlighting the importance of a conserved inner Core-OS structure in this family. While no differences were found for *wa* Region 3—the genes *waaC* and *kdkA* were found highly conserved next to each other across all *Aeromonas* genomes studied—we identified four different genomic organizations regarding *wa* Region 2.

**Figure 2:**
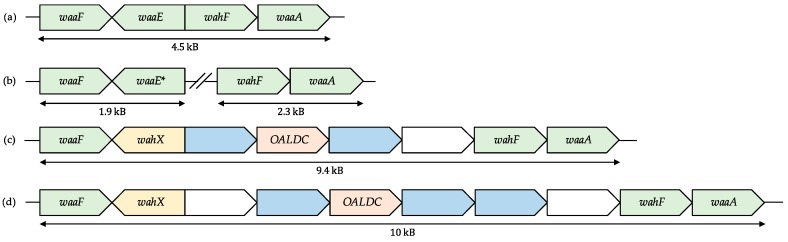
Comparison of the *wa* Region 2 in *Aeromonas*. (**a**) The most common genomic organization for this region found in the majority of *Aeromonas* species, with *waaF*, *waaE*, *wahF*, and *waaA* positioned sequentially in a gene cluster; (**b**) In the species *A*. *diversa*, *A*. *schubertii*, and *A*. *simiae*, this cluster is divided in two different genomic regions. Moreover, *A*. *simiae* presents *wahX* instead of *waaE*, marked with an asterisk; (**c**) A gene insertion of six genes containing *wahX*, a gene coding for an O-antigen ligase domain-containing protein (OALDC, in orange), and two glycosyltransferases (in blue) between *waaF* and *wahF* is found in the species *A*. *bivalvium*, *A*. *molluscorum*, and *A*. *rivuli*. Hypothetical proteins with unknown function are shown in white; and (**d**) Similarly, in the *A*. *fluvialis* genome, there is a gene insertion of seven genes containing *wahX*, a gene coding for an O-antigen ligase domain-containing protein, and three glycosyltransferases between *waaF* and *wahF*.

Overall, the synteny of the *wa* Region 2 gene cluster was predominantly conserved across most of the *Aeromonas* species, with *waaF*, *waaE*, *wahF*, and *waaA* positioned sequentially, as we previously described for *A*. *piscicola* AH-3 and *A*. *salmonicida* (Figure 2).

In the sequenced genomes of *A*. *schubertii*, *A*. *diversa*, and *A*. *simiae* strains, these genes were found split in two regions: *waaF* and *waaE* on one genomic site (*wa* Region 2.1); and *wahF* and *waaA* in another (*wa* Region 2.2) (Figure 2). It is worth mentioning that a fully closed genome from a species with this alternative genomic organization is not publicly available and, given the position of these regions in the ending part of unassembled contigs, we cannot rule out that this genomic distribution may be caused by an artefact in the genomic assembly. Although we did not detect major differences in the gene sequences of *A*. *schubertii* and *A*. *diversa*, the highly-conserved glycosyltransferase *waaE* is substituted in *A*. *simiae* by a gene which translated protein shows the same β-1-4-glucosyltransferase domain but less than 30% aminoacidic homology with WaaE. We named this gene, found in *Aeromonas* for the first time, *wahX*. The number of appearances and degree of conservation of *wahX* in *Aeromonadaceae*—together with the rest of genes discussed in this article—can be found in the Table 1.

The gene *waaE* codes for the l-glycero-d-manno-heptose β-1,4-d-glucosyltransferase, which catalyses the union of a Glc residue to the highly conserved l-α-d-HepI of the inner Core-OS not only in *Aeromonas* species (as *A*. *piscicola* [8] and *A*. *salmonicida* [9]), but also in other *Enterobacteriaceae* as *Klebsiella pneumoniae* and *Serratia marcescens* [10]. Thus, the substitution of this gene inside the *Aeromonadaceae* family is striking. Instead of with the rest of *Aeromonas* species, the protein sequence of *wahX* displays high homology with glycosyltransferases of other *Proteobacteria*, as *Methylomarinum* and *Desulfovibrio*. The chemical composition of the *Desulfovibrio desulfuricans* LPS polysaccharide chain was recently revealed [11], containing residues of Kdo, rhamnose, methylopentose-fucose, 3 hexoses-mannoses, glucose, and galactose, but the structure of this bacterium Core-OS chain, and thus the exact function of *wahX*, remains unknown for the time being.

We also detected major changes in the *wa* Region 2 of *A*. *fluvialis*, as well as in the evolutionary-related group of *A*. *bivalvium*, *A*. *molluscorum*, and *A*. *rivuli*. As in *A*. *simiae*, these species presented *wahX* instead of *waaE*. Furthermore, an insertion of six genes in *A*. *fluvialis* and four genes in *A*. *bivalvium*, *A*. *molluscorum*, and *A*. *rivuli*, between *wahX* and *wahF* was found in these genomes (Figure 2).

The four different subgroups of *Aeromonas* species as categorized by their *wa* Region 2 genomic organization correlate well with the asumed evolution of this genus. Colston et al. recently compared the genomes from 56 different strains to revise and improve the phylogenetic and taxonomic relationships between *Aeromonas* species [12]. Despite that the evolutionary reconstruction of the genus is still not completely resolved and varies depending on the gene set used for the phylogeny, their results clearly defined of eight major monophyletic groups inside *Aeromonas*. Supporting our findings, *A*. *schubertii*, *A*. *diversa*, and *A*. *simiae* on one side and *A*. *bivalvium*, *A*. *molluscorum*, and *A*. *rivuli* on the other were precisely classified as two of these eight major *Aeromonas* monophyletic groups.

However, the resemblance between alternative *wa* Region 2 Types 3 and 4 despite their differential evolutionary position is intriguing. While *A*. *bivalvium*, *A*. *molluscorum*, and *A*. *rivuli* are well-defined evolutionary-related species [13], the *A*. *fluvialis* genomic sequence is more related to species of the *A*. *veronii* monophyletic group (see [12]) both by DNA-DNA hybridization [14] and by in silico whole genome analyses [12]. Therefore, an in-depth study of horizontal gene transfer between these species may be warranted.

### 2.2. Synteny of the wa Region 1

Regarding *wa* Region 1, at least two glycosyltransferases were located next to *tetR* and *hldD* in all genomes. The *A*. *diversa* 2478-85, *A*. *lacus* AE122, *A*. *salmonicida subsp*. *achromogenes* AS03, and *A*. *veronii* VBF557 genomes were excluded from the analysis as the assembly of this region in these species was broken between different contigs. Thus, 166 genomes were analysed. Overall, we found considerable variability in the outer core genes of the *wa* Region 1 cluster across the available *Aeromonas* genomes, with at least 29 different genomic organizations containing different genes, based in their sequence homology, in the 166 analysed genomes. The complete assignment of *wa* Region 1 types to each strain can be found at the Table S1.

**Figure 3:**
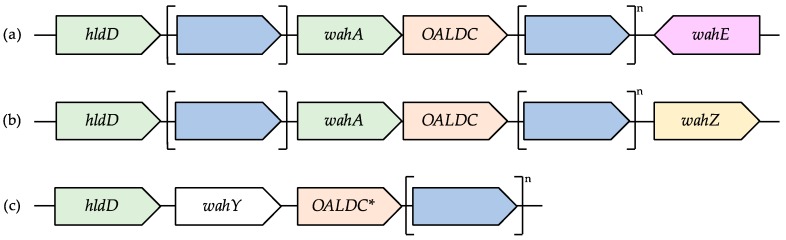
Schematic description of the different *Aeromonas wa* Region 1 types reported. (**a**) The gene *hldD* was found at the start of the *wa* Region 1 in all *Aeromonas* species. In *A*. *salmonicida*, a glycosyltransferase-coding gene (in blue) is present between *hldD* and *wahA*. A gene coding for an O-antigen ligase domain-containing was always found next to *wahA*, followed by a variable number of hypothetical genes and glycosyltransferases, and, finally, *wahE*; (**b**) Most of the species of the *A*. *veronii* monophyletic group present the same genomic architecture but with *wahZ* instead of *wahE*; and (**c**) In the species in which genome *wahA* is not present, *wahY* is found next to *hldD*, followed by a gene coding for an O-antigen ligase domain-containing protein (except in the species described above where this gene is found in the *wa* Region 2, marked with an asterisk) and a variable number of glycosyltransferases and hypothetical proteins. OALDC = O-antigen ligase domain containing gene.

While the description and characterization of each of the outer LPS core biosynthetic clusters expands far beyond the scope of this article, we can provide a rough set of patterns describing the *Aeromonadaceae wa* Region 1 derived from our genome analysis, which are summarized in Figure 3. First, the *wa* Region 1, containing *hldD*, at least two glycosyltransferases and one gene coding for a protein with an O-antigen ligase domain (i.e., *waaL* in *A*. *piscicola* and *A*. *salmonicida*), was found upstream of *tetR*. We found two exceptions to this pattern: the *A*. *media* strains CECT 4232 and WS, which showed only one glycosyltransferase (*wahY*, characterized below) instead of more; and the species *A*. *fluvialis*, *A*. *bivalvium*, *A*. *molluscorum*, and *A*. *rivuli*, in which this genomic region was composed only by *hldD* and one glycosyltransferase (*wahY*), while the O-antigen ligase-domain containing gene was located in the previously mentioned genomic insertion of the *wa* Region 2 (Figure 2).

Despite presenting high sequence variability between species, all the O-antigen ligases from the different *wa* Region 1 genomic organizations shared 10 aminoacid positions that appear to be critical for its function: four glycines, three arginines, one serine, one tryptophan and one histidine residues (positions 123, 196, 245, 264, 268, 272, 286, 288, 318, and 329 for *A*. *piscicola* AH-3 WaaL) (Figure S1a). Furthermore, all putative O-antigen ligases presented between 8 and 11 predicted transmembrane domains, with 10 being the most common prediction (Figure S1b), as is characteristic for lipid-A-core, O-antigen ligases [8,15].

The second pattern that we found is the high conservation of WahA, the protein that links GlcNAc to the LPS outer Core-OS using UDP-GlcNAc as a substrate. A gene coding with a protein with high homology to *wahA* was found in 153 of the 166 *Aeromonas* genomes. It was absent in the genomes of *A*. *bivalvium* CECT 7113, *A*. *caviae* AE398, CECT 4221, and FDAARGOS_76, *A*. *fluvialis* LMG 24681, *A*. *media* CECT 4232 and WS, *A*. *molluscorum 848*, *A*. *rivuli* DSM 22539, *A*. *salmonicida* Y47, *A*. *simiae* CIP 107798, and *A*. *veronii* AVNIH1 and AVNIH2. Interestingly though, all these genomes shared the presence of a common glycosyltransferase next to *hldD* with high homology to others from *Vibrio coralliilyticus*. We named this gene *wahY*, and it was not present in any of *Aeromonas* genomes with *wahA*. Interestingly, *wahY* was presend in all the species with a *wa* Region 2 of the newly-described Types 3 and 4.

The importance of WahA remains in its bifunctional nature: it has two domains, one glycosyltransferase domain that catalyses the incorporation of GlcNAc to the LPS outer Core-OS; and one carbohydrate esterase domain deacetylates the GlcNAc residue to GlcN [16]. The gene *wahA*, is not only conserved in *Aeromonas*, but also in *Vibrio cholerae* and *shilonii* species. Thus, its substitution for *wahY*, which is annotated as type I glycosyltransferase in the online databases but shows no protein domains, technically negates the characteristic presence of GlcN in the outer Core-OS of these species.

Thirdly, the presence in the *wa* Region 1 of all genomes with *wahA* of a glycosyltransferase belonging to the inner LPS Core-OS biosynthesis. More importantly, when present in the genome, this gene was always found delimiting the end of the *wa* Region 1 cluster. We found two different gene sequences based on their homology: those that displayed high homology with *wahE* and were positioned contrary to the *hldD* direction; and those that displayed high homology to an uncharacterized glycosyltransferase, which we named *wahZ*, and were positioned in the same direction as *hldD*. Given the exclusivity of each of these genes in the genomes, we hypothesize that both genes code for a protein that catalyse the union of a carbohydrate (HepIV in the case of *wahE*) in the same position of the l-α-d-HepI.

An homolog of *wahE* was found on 101 of the 153 genomes with *wahA*, comprising the species *A*. *aquatica*, *A*. *bestiarum*, *A*. *caviae*, *A*. *dhakensis*, *A*. *encheleia*, *A*. *enteropelogenes*, *A*. *eucreophila*, *A*. *hydrophila*, *A*. *jandaei*, *A*. *media*, *A*. *piscicola*, *A*. *popoffii*, *A*. *salmonicida*, *A*. *sanarellii*, *A*. *schubertii*, *A*. *taiwanensis*, *A*. *tecta*, and some undefined *sp*. strains; while *wahZ* was found in 52 of the 153 genomes, comprising the species *A*. *allosaccharophila*, *A*. *australiensis*, *A*. *diversa*, *A*. *enteropelogenes*, *A*. *finlandiensis*, *A*. *jandaei*, *A*. *sobria*, *A*. *veronii*, and a few undefined *Aeromonas sp.* strains. In consequence, the presence of *wahZ* seems to be a defining characteristic of the *A*. *veronii* monophyletic group described by Colston et al. [12].

#### Most Common *wa* Region 1 Genomic Organizations and Notable Inconsistencies

**Figure 4:**
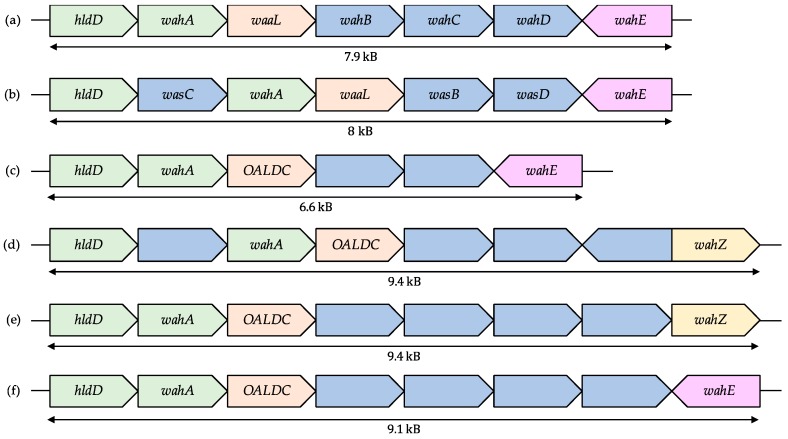
Comparison of the six most common *wa* Region 1 genomic organization in *Aeromonas*. Common genes (*hldD* and *wahA*) in these genomic organizations are shown in green, O-antigen ligase domain containing proteins in orange, *wahE* in purple, *wahZ* in yellow, and both characterized and uncharacterized glycosyltransferases are shown in blue. (**a**) Type 1 *wa* Region 1, found in *A*. *bestiarum*, *A*. *dhakensis*, *A*. *hydrophila*, *A*. *piscicola*, and *A*. *schubertii*; (**b**) Type 2 *wa* Region 1, found in *A*. *salmonicida* species; (**c**) Type 3 *wa* Region 1, as in *A*. *aquatica*, A. *caviae*, *A*. *media* and *A*. *sanarelli*; (**d**) Type 4 *wa* Region 1, found in *A*. *australiensis*, *A*. *jandaei*, and *A*. *veronii*; (**e**) Type 5 *wa* Region 1, in *A*. *enteropelogenes* and *A*. *finlandensis*; and (**f**) Type 6 *wa* Region 1, as found in *A*. *popoffi*.

A schematic representation of the six most common gene organizations for *wa* Region 1, depicting 122 of the 170 genomes, can be found at Figure 4. The most common *wa* Region 1 genomic structure, or Type 1, was the one described for *A*. *piscicola* AH-3 [8]. We found this organization in the genomes of the species *A*. *bestiarum*, *A*. *dhakensis*, *A*. *hydrophila*, *A*. *piscicola*, and *A*. *schubertii*. The second type of *wa* Region 1 (Type 2) that we found was that of *A*. *salmonicida*, including the subspecies *masoucida*, *salmonicida*, and *smithia*, but not *pectinolytica* or *achromogenes*. A group of *A*. *caviae* and *A*. *media* species, together with *A*. *aquatica* and *A*. *sanarelli* also showed common genes for this genomic region, which we assigned Type 3. The second most common genomic organization for this cluster, Type 4, comprised 23 different *A*. *veronii* strains, *A*. *australiensis*, and *A*. *jandaei*. The Type 5 *wa* Region 1—assigned to the genomic cluster found in *A*. *enteropelogenes* and *A*. *finlandensis* species—and Type 6—in *A*. *popoffi*—highlight the high recombination capacity of this region in the genus: both types present the same genomic architecture and high homology percentage between the genes, but *wahZ* is found in the Type 5 while *wahE* is found in the Type 6 *wa* Region 1.

Our results at the genomic level largely agree with the presumed evolution of the *Aeromonas* genus, and are corroborated by the published reports regarding LPS structure in the aforementioned species. For example, *A*. *bestiarum* presents the same *wa* Region 1 Type 1 than *A*. *hydrophila* and *A*. *piscicola*, and the mass spectrometry of the Core-OS from *A*. *bestiarum* Strain K296 (serotype O18) shows the identical Hep_6_Hex_1_HexN_1_Kdo_1_P_1_ structure that we characterized for *A*. *piscicola* AH-3 and *A*. *hydrophila* AH-1 [7,17]. Similarly, although the genome of the strain *A*. *veronii* strain Bs19 (serotype O16), is not available, the LPS chemical structure of this strain is Hep_5_Hex_3_HexN_1_Kdo_1_P_1_, that is, one more carbohydrate than the ones described for the Type 1 *wa* Region 1 [18]. Coinciding, the *wa* Region 1 of the majority of *A*. *veronii* (Type 4) is composed by one more gene than the ones described for Type 1. Moreover, given the chemical structure, we hypothesize that *wahZ* catalyses the union of a hexose to HepI.

However, the previous description of these *wa* Region 1 organizations also arise notable inconsistencies between taxonomic classifications in some species. Beyond the species mentioned above, the *wa* Region 1 Type 1 was also found the strains *A*. *enteropelogenes* LK14 (that according to the rest of *A*. *enteropelogenes* strains should be of Type 5), *A*. *jandaei* L14h (Type 4), and *A*. *salmonicida* strains CBA100 and *subsp*. *pectonolytica* 34meI (both Type 2). Of note, we previously pointed out that *A*. *salmonicida subsp*. *pectinolytica* presented a different outer LPS Core-OS than the rest of *A*. *salmonicida* species and similar to *A*. *hydrophila*, supporting this exception [19].

Similarly, *A*. *hydrophila* 4AK4 and *A*. *hydrophila* BWH65 present a Type 3 *wa* Region 1 instead of the Type 1 common for the rest of *A*. *hydrophila* species. *A*. *hydrophila* 4AK4 shows extreme resemblance to *A*. *caviae* (also Type 3 for some strains) in many areas, such as the—almost exclusive in aeromonads—production of poly(3-hydroxybutyrate-co-3-hydroxyhexanoate) [20] and the higher sequence homology of virulence factors to *A*. *caviae* than *A*. *hydrophila* [21]. In fact, *A*. *hydrophila* 4AK4 shows low average nucleotide identity (ANI) values with *A*. *hydrophila* and has been proposed to be a novel species relative to *A*. *media* [22], also represented in the Type 3 *wa* Region 1 organization. Likewise, *A*. *hydrophila* BWH65 shows high genome homology with *A*. *hydrophila* 4AK4, and both are found in the same branch as *Aeromonas* SSU (recently reclassified as *A*. *dhakensis* SSU) in the genomic blast-based dendogram of the *A*. *hydrophila* webpage in the NCBI genomes (Available online: https://www.ncbi.nlm.nih.gov/genome/1422).

Of note, notable variability between these regions was found in the species *A*. *veronii*, *A*. *caviae*, and *A*. *media*, which may indicate a higher recombination capacity or taxonomic nomenclature inconsistencies in this species.

### 2.3. Reconstruction of the Common Aeromonas Inner LPS Core-OS

**Figure 5:**
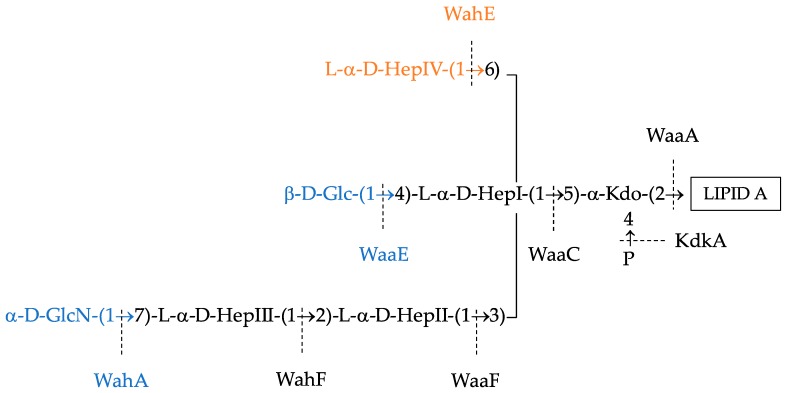
Reconstruction of the *Aeromonadaceae* common Core-OS. The enzymes found in all genomes are shown in black. WaaE and WahA (in blue), were also present in more than the 90% genomes analysed. Finally, WahE (in orange) is substituted by WahZ (although the carbohydrate linked is still unknown) in the species of the *A*. *veronii* monophyletic group.

Using the above information, we could reconstruct a common LPS inner core in the entire *Aeromonas* genus composed by three l-α-d-Heptoses and one α-Kdo-P, based on the conserved genes *kdkA*, *waaA*, *waaC*, *waaF*, and *wahF* (Figure 5). Moreover, with the exception of the species *A*. *bivalvium*, *A*. *molluscorum*, *A*. *rivuli*, *A*. *fluvialis*, and *A*. *simiae*, *waaE* was also found in all genomes. Similarly, only in eight strains (*A*. *caviae* strains AE398, CECT4221, and FDAARGOS76; *A*. *media* strains CECT 4232 and WS; *A*. *salmonicida* Y47; and *A*. *veronii* strains AVNIH1 and AVNIH2) more than in the aforementioned five species *wahA* was not found in the genome—all of them presented *wahY*. Thus, we consider *waaE* and *wahA* as highly conserved genes in aeromonads, and in consequence a high conservation of the hexose β-1,4-Glc linked to HepI, and of the α-1,7-GlcN linked to HepIII in the aeromonad LPS core (Figure 5). Furthermore, we found *wahE* to be well conserved across most of *Aeromonas* species except in the *A*. *veronii* monophyletic group, which presented the gene *wahZ* instead, suggesting that they may act in the carbohydrate same position.

**Figure 6:**
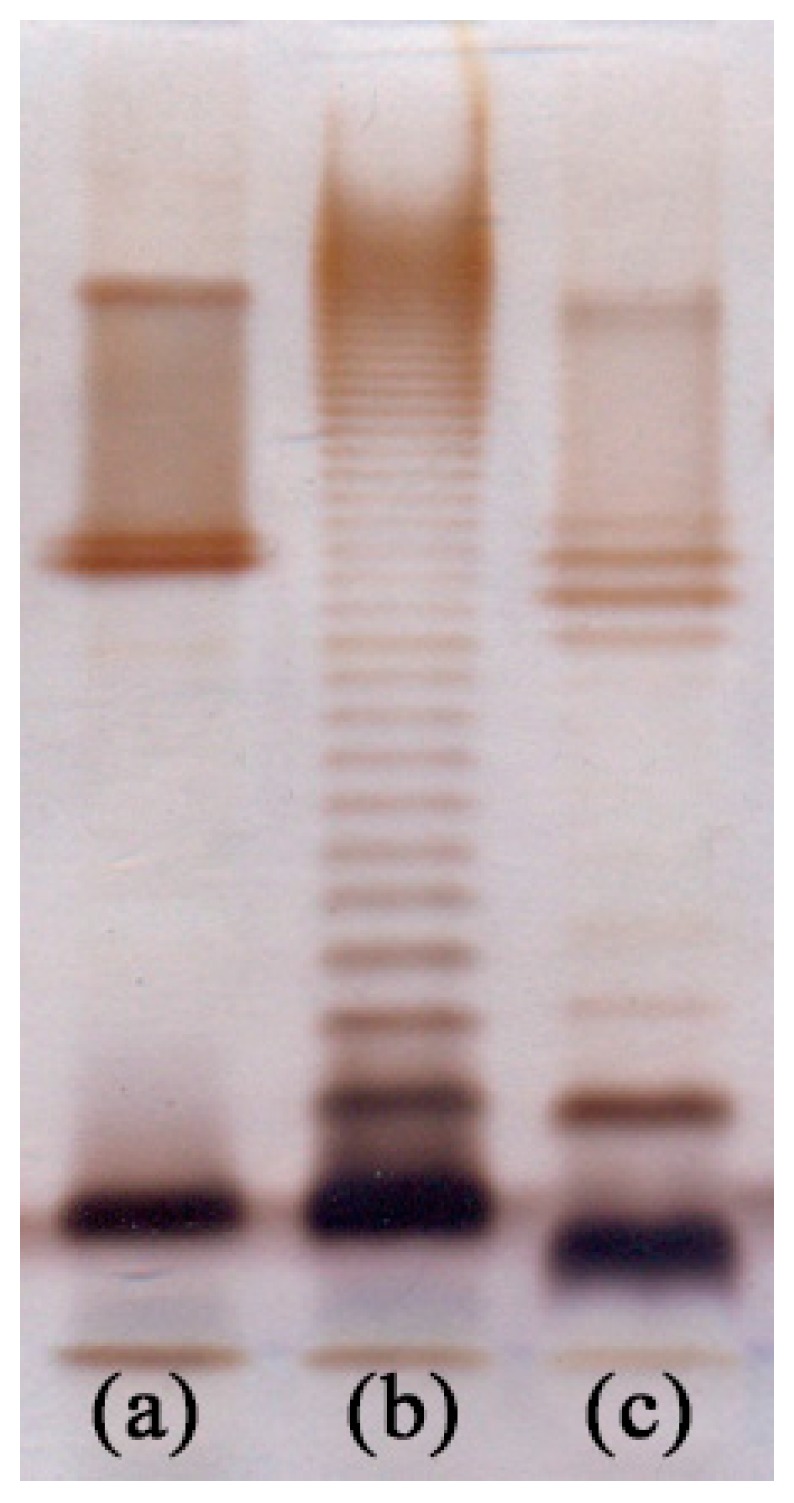
LPS profiles of (**a**) *A. hydrophila* AH-1; (**b**) *A. piscicola* AH-3; and (**c**) *A. bivalvium* 868E^T^ by SDS-PAGE.

Finally, it is worth mentioning that the absence of typical outer core homologs (as *wahB*/*wasB*, *wahD*/*wasD*) and *waaE* in *A*. *bivalvium*, *A*. *fluvialis*, *A*. *molluscorum*, *A*. *rivuli*, and *A*. *simiae*, suggests that either these species do not present O-antigen or their whole outer Core-OS may be entirely different than in the rest of *Aeromonas*. The presence O-antigen ligase domain-containing protein with similar homology in the *wa* Region 1 on *A*. *simiae* and *wa* Region 2 gene insertions of *A*. *bivalvium*, *A*. *fluvialis*, *A*. *molluscorum*, and *A*. *rivuli* supports the latter. To prove the presence of O-antigen in these species, we analysed the LPS profile gel of the species *A. hydrophila* AH-1, *A. piscicola* AH-3, and *A. bivalvium* 868E^T^ by SDS-PAGE (Figure 6). The LPS profile gel confirmed the presence of O-antigen in *A. bivalvium* (and therefore, probably in the other species questioned) as well as a considerable difference in the LPS Core-OS size.

## 3. Discussion

*Aeromonas* are a ubiquitous, rod-shaped and flagellated genus of Gram-negative bacteria emerging as important animal pathogens, especially in mammals and fish. Despite once being subdivided into solely two subgroups, mesophilic (*A*. *hydrophila*) and psychrophilic (*A*. *salmonicida*) aeromonads [1], there are currently dozens of characterized *Aeromonas* species in the literature, not including subspecies or biovars (Available online: http://www.bacterio.net/aeromonas.html for a complete list).

However, the taxonomic and phylogenetic relationships of many of these species are still today a matter of debate: practical evidence for some of these species is still lacking [1]; different names have been found to be synonyms for the same species (e.g., *A*. *enteropelogenes* and *A*. *trota* [23,24]; and *A*. *culicicola*, *A*. *ichthiosmia* and *A*. *veronii* [23]); characterized species have been reclassified outside the *Aeromonas* genus (as *A*. *sharmana* [25]); and, historically, misclassifications in this family have been usual (e.g., *A*. *hydrophila* AH-3 was recently reclassified into *A*. *piscicola* AH-3 [26]). This taxonomic classification problem is further exacerbated by a considerably high genetic homologous recombination rate and horizontal gene transfer capacity, which in fact has been demonstrated to be an important driving force in the evolution of aeromonads [27]. When taking all of this together, it is not difficult to understand why the evolutionary reconstruction of this family is still not resolved. The importance of a correct classification of *Aeromonas* species resides not only in correctly knowledge of taxonomic evolution, but also in studying the pathogenic source and potential of *Aeromonas* species and strains.

As the virulence of *Aeromonas* species is multifactorial and depends on the specific virulence factors present on each strain as well as on environmental conditions [4], the description of common processes governing aeromonad pathogenicity is of crucial importance. The LPS, an important component of the Gram-negative membrane present in all aeromonads, also plays a key role in the adhesion and infectivity process, thus directly affecting their pathogenicity. Therefore, the study of the specific LPS biosynthesis routes and structure may be further exploited in, for example, the search for common treatments against *Aeromonas* species or specific inhibitors for characteristic strains. To this end, in this study, we compared the genomic structure of the three described locus for the LPS Core-OS biosynthesis—*wa* Regions 1, 2, and 3—in all publicly available *Aeromonas* genomes, covering 170 strains spawning 30 different *Aeromonas* species to study their degree of conservation.

As expected, the genes and genomic organizations of the inner core regions were more evolutionary conserved than the outer core region, but adding to our previously characterized *wa* Region 2 in *A*. *piscicola* and *A*. *salmonicida*, we describe three new genomic organizations for these regions affecting the species *A*. *bivalvium*, *A*. *diversa*, *A*. *fluvialis*, *A*. *molluscorum*, *A*. *rivuli*, *A*. *schubertii*, and *A*. *simiae*.

In contrast, the outer Core-OS biosynthetic region presented remarkable variability within *Aeromonas*. In fact, *hldD* (formerly *rfaB*, coding for ADP-l-glycero-d-manno-heptose 6-epimerase) was the only common gene of the *wa* Region 1 that we could locate in all analysed genomes. Therefore, we consider *hldD* as a good marker for *wa* Region 1 position. Despite the differences between species, we also found a gene coding for a O-antigen ligase domain-containing protein and at least two glycosyltransferases in the *wa* Region 1 of all *Aeromonas* species except *A*. *bivalvium*, *A*. *fluvialis*, *A*. *molluscorum*, and *A*. *rivuli*. We also detected high degree of conservation of *wahA* (in 154 of the 166 genomes analysed for *wa* Region 1), and the common presence of *wahY* in the 12 species lacking this gene (Table 1). Similar exclusivity patterns were found between *wahE* and *wahZ*, which appears to be an exclusive marker of the *A*. *veronii* monophyletic group. Furthermore, our results are supported by both genome-wide phylogenetic and chemical mass spectrometry studies in the literature.

We report that, while horizontal gene transfer—not involving only *Aeromonas* species but also other proteobacteria—seems to be a potential key process for the evolution of this regions, the genes of the LPS Core-OS are considerably well conserved throughout *Aeromonas* evolution. Therefore, these genomic regions can be useful when studying the taxonomic relationships between *Aeromonas* species.

Our study found notable inconsistencies between the conservation of the genomic organization and the taxonomy of specific strains, such as *A*. *enteropelogenes* LK14, *A*. *hydrophila* strains 4AK4 and BWH65, *A*. *jandaei* L14h, and *A*. *salmonicida* strains CBA100 and *subsp*. *pectonolytica* 34meI. Given that extensive scrutiny of at least two of these strains arise important criticism and suspicion about their taxonomic classification, an in-depth study of this species taxonomic classification may be necessary.

Finally, the recent reclassifications of several *Aeromonas hydrophila* species, as *A*. *hydrophila* AH-3 (now *A*. *piscicola* AH-3), led recently to a serious questioning if previous research involving pathogenic factors on strains of now different *Aeromonas* species was still applicable to *A*. *hydrophila* [28]. The results from this study support that the LPS Core-OS from *A*. *hydrophila* is identical to that of *A*. *piscicola*, and thus previous research regarding this virulence factor should be applicable to *A*. *hydrophila*.

## 4. Materials and Methods

We retrieved the 170 different genomes for the *Aeromonas* genus available on NCBI Genome website as of September 2016 (Table S1), regardless of their assembly completeness. To roughly locate the position of the LPS core biosynthetic regions in the genomes, we performed a local tblastn of *A*. *hydrophila* ATCC7966 *tetR* (*wa* Region 1), *waaA* (*wa* Region 2), and *waaC* (*wa* Region 3) in each genome assembly. Due to the incomplete annotation of some of the analysed genomes, the 16,000 bp region upstream of *tetR*, 10,000 bp region upstream of *waaA*, and 5,000 bp region downstream of *waaC* were reannotated in each genome. Gene prediction was performed with Glimmer v.3.0.2 [29] and hand-curated. Comparison of the genetic regions was performed using the CloVR Comparative Pipeline [30] and explored using Sybil [31].

The predicted protein sequence of one arbitrarily selected O-antigen ligase from each *wa* Region 1 genomic organization was retrieved and aligned with MUSCLE [32]. Prediction of the transmembrane domains was performed with TMHMM Server v2.0 [33].

Cultures of *A*. *hydrophila* AH-1, *A*. *piscicola* AH-3 and *A*. *bivalvium* 868E^T^ where grown overnight in Tryptic Soy Agar at 30 °C, except for *A. piscicola* AH-3 which was grown at 25°C. LPS was obtained after proteinase K digestion of whole cells, separated by SDS-PAGE and visualized by silver staining as previously published [16].

## 5. Conclusions

We compared the LPS biosynthetic regions from 170 *Aeromonas* genomes and analysed the conservation of the genes taking part in the assembly of this cell-wall component. We describe high conservation of the genes related to the inner Core-OS biosynthesis, with only one organization for *wa* Region 3 and 4 different genomic organizations regarding the *wa* Region 2; and remarkable variability in the *wa* Region 1, composed roughly by genes affecting the outer Core-OS. Besides describing a common LPS Core-OS structure of all the *Aeromonas* sequenced to date, we also report how these regions can be useful for establishing evolutionary relationships between species.

## Figures and Tables

**Table 1:** Common genes of the *wa* Regions 1, 2, and 3, and their conservation in the analysed genomes.

*wa* Genomic Region	Gene	Number of Genomes in Which Appears	Conservation in Studied *Aeromonas*
1	*hldD*	166/166	100%
	*O-antigen ligase*	166/166	100%
	*wahA*	154/166	93%
	*wahY*	13/166	7%
	*wahE*	101/166	61%
	*wahZ*	52/166	31%
2	*waaA*	170/170	100%
	*wahF*	170/170	100%
	*waaE*	165/170	97%
	*wahX*	5/170	3%
	*waaF*	170/170	100%
3	*waaC*	170/170	100%
	*kdkA*	170/170	100%

## Supplementary Materials

Supplementary materials can be found at www.mdpi.com/1422-0067/18/3/519/s1.

## Abbreviations

LPS	Lipopolysaccharide
Core-OS	Core-OligoSaccharide
Kdo	3-Deoxy-d-manno-oct-2-ulosonic acid
OALDC	O-antigen Ligase Domain Containing gene
ANI	Average Nucleotide Identity
