# Supplementary material for: Comparative Genomics of the Aeromonadaceae Core Oligosaccharide Biosynthetic Regions
Source: Int J Mol Sci. 2017 Feb 28;18(3):519. doi: 10.3390/ijms18030519 (PMC5372535; doi:10.3390/ijms18030519)
Supplement: Supplementary file 1 [file ijms-18-00519-s001.zip › Supplementary Figure 1.pdf]

Supplementary Figure 1(a). Alignment of the O-Antigen ligase domain-containing predicted proteins for each of the types assigned for *wa* Region 1.

```
olc1_piscicola_ --------------------MPIRCLYL--------------LDKTTTALFALT-----I
olc2_salmonicid --------------------MSIRSLEL--------------LDKATTALFALV-----I
olc22_tecta     --------------------MSIKPLEI--------------LDKVTTAVFALT-----I
olc4_veronii_Hm --------------------MAQNSRKLCLQEIKVISKNAISMERFALLIFSCT-----L
olc5_popoffi    ----------------------------------------------------MT-----L
olc6_enteropelo ----------------------MNMTQY--------------FHRLNVLIFSCT-----L
olc21_taiwanens --------------------MPINSKIL--------------LVRMTIGLFSLS-----I
olc20_veronii_A ----------------------------------------MTIEKVTEYFFALT-----I
olc11_veronii_A MI------------------TICKNKKI--------------VSYMLHAYYLLP-----M
olc25_sp_ZOR000 -----------------------------------------------------------M
olc7_veronii_B5 --------------------MAICSPLI-----------MPFRSHLTHNLVQTS-----A
olc12_caviae_42 --------------------MILASPII-----------LPLRINILHNLVQVS-----A
olc13_media_423 --------------------MTLASPII-----------LPLRINILHNLVQVS-----A
olc14_media_ws  --------------------MTLASPII-----------LPLRINILHNLVQVS-----A
olc15_allosacch ------------MFLGNKLFMTPTHNIF-----------LSWRDNATHNLVQVG-----A
olc9_allosaccha ------------------------------------------------------------
olc19_sobria_42 ------------------------------------------------------------
olc24_salmonici ------------------------------------------------------------
olc23_145       VT-------------------------------------SYKKYLRLNIIVQFA-----A
olc17_jandaei_4 ---------------------MQEIMMV-----------LFSKEKFVPFMVQAS-----A
olc18_sp_EERV15 MCSGCGVKSVGIMFLES---EKRKRMMV-----------LVNKERFVPLVVQTS-----A
olc26_encheleia ------------MYLGSK--MYPTISII-----------PTKRAELLPWLVQLS-----A
olc8_bv_sobria_ MFSENNVAVEKQMSLEKN--PTIIGSHF-----------FLNRRNIFPLLVQLS-----A
olc10_caviae_83 MSSENKVVVEERMPMQTE--SPPNQGAF-----------GLVHDQALPLLVQLS-----A
olc28_caviae_42 ------------------------------------------------------------
olc3_caviae_A23 --------------MTDH--DSSTSDYFL--------R-----------IFHGQAFGNFI
olc27_simiae    MLL--------LDIFKSH-W-------------------PSGRRAQVESVLRYGGLAC-L
olc16_diversa   --------------MFSE--NEKKSTGF----------NCLMIEKILVGIFSAS-----I
olc29_veronii_1 ----------------------MKEKKM----------NYKKIETYLLCLLAAS-----I


olc1_piscicola_ IF----SFCGLFLVPAGQSIESNLLAVTGIFGLL---NYFVGKQQDVGLKD--RRILWGL
olc2_salmonicid LF----SFCGLFLVPAGQTILSNLLVVASVFGLL---NYFVGKKRDVGLED--RRILWVL
olc22_tecta     AF----SFCGLFLVPSGQSILSNLLVVSGVLGLL---NYFIGGKKEVLFTD--RRLIWIF
olc4_veronii_Hm IF----AFSVMFFFTDGKTYLSNLIVVASIIGMV---SFYI-HRYPVGFKN--RHIIWVL
olc5_popoffi    FF----TLCGLFIVPLGQHYLTKLLVLSSIIGVI---NYFVGNKELIGVSA--KNLYWCV
olc6_enteropelo FF----TLCGLFLVPQGNHYLSKLLVLSSIVGFI---NYFIGNKDKLGVSA--KNLYACV
olc21_taiwanens IF----LFCGLFIVPSGTSYMSTLIVLSCFFGVV---NFFTGDKTSVGLRD--KRLLFAF
olc20_veronii_A FF----AFGFLYASARGHSYLSIFVVISIILGAI---NLIY-NKEIGTLKSGCGFLLKIT
olc11_veronii_A LI----LSCLAFNTEDGKHYISRLVVAMFIISLFTARKILWSNLNNPEI----KRIMFFW
olc25_sp_ZOR000 LI----LSCLTFTTFDGKHYISRLVVVMFVISCVFCRNTIINNIKKAEV----RRILFFG
olc7_veronii_B5 FF----FGALCLAIPSGYSYGPVLLSIMSLFVFW-RADYWLAMSREA------KILFWIF
olc12_caviae_42 FL----FGALALTIPSGYSYGPAILLLASITVCW-RPFYWVNMPREV------KILALIF
olc13_media_423 FL----FGALTLAIPSGYSYGPAILLLASLTVCW-RPSYWMNMPREV------KILALIF
olc14_media_ws  FL----FGALTLAIPSGYSYGPAILLLASLTVCW-RPSYWMNMPREV------KILALIF
olc15_allosacch FL----FGALSLAIPSGYSYGPAILLAVSLTVCW-RPLYWLRMPKEV------KMLALFF
olc9_allosaccha ------------------------------------------MSSEV------KILSYIF
olc19_sobria_42 ------------------------------------------MSSEA------RILSYIF
olc24_salmonici ------------------------------------------MSSEV------KILSYIF
olc23_145       FL----FGAIVLTVPSGYGYGPSILVLASFFVFW-RKKYYLAMSSEA------KTLLYIF
olc17_jandaei_4 FL----FGALALAVPSGYSYGPALLAVVSLFVFW-RKDYLSAMEKEH------KIIASLL
olc18_sp_EERV15 FL----FGALALAVPSGYSYGPALLAIVSLFVFW-RKEYLSAMEKEH------KIIASLL
olc26_encheleia FS----FGALAIAIPSGYSYGPALLAVISLFVFW-RKEYLDCLTKEH------KVVAALL
olc8_bv_sobria_ FL----FGALALTMPSGYSYGPGLLLLAGLGFIICKRSSALSLPFEL------KIISLAF
olc10_caviae_83 FL----FGALALSVPSGYSYGPALLLLASLCFFAVRRLNWV-LPREL------KFIGLGL
olc28_caviae_42 ------------------------------------------MPREL------KLIGLGL
olc3_caviae_A23 FLNILGYTLFKDSIQLIADVCQTIIVLATLMAFYVERAFFTRNRIF-------QLLCLAL
olc27_simiae    FL----YALSRLALPGLGKGLEIGYVLAGAILLFGWRRELLGSIYI-------KLLLLAL
olc16_diversa   FV-----------SKPAITISMALLVGFSLFRYFKDGEYRSALRG--------DAIVVSS
olc29_veronii_1 FT-----------SKPAIYISSALMIVFFIGRCIFDRDYYNVVKR--------NIVLWGP
                                                                       :
```

```
olc1_piscicola_ VVYAAMIFVNRLI---HGDQYGVMRGLFYVVIFALMMP------RKPILLMLGYLAIVLG
olc2_salmonicid AAYAAMIFVNRLI---HGDQYGVMRGLFYVVVFALMIP------RKPVLLTLGYAAIVTG
olc22_tecta     VFYATVILINRII---HGDQYGVMRDLFYVSVFSLMMP------RKKILFSVGCIAILIG
olc4_veronii_Hm LFYAIYLFVNRQI---HGDQYGIMRAIIYVIFFTFLIP------RNVFLLKAGCYGVILG
olc5_popoffi    LIYIVAIGISRLV---HDDESWLIRNLLYILAFFLLMP------REAVLVSVFKLGSLVG
olc6_enteropelo LIYIAAIGASRLV---HDDESWLIRNLFYILVFFLFIP------REAIVIRALKLGALIG
olc21_taiwanens LAYALVILVNRQI---HGDASWIMRNILYVIVFSFFLP------RERIIINIGVLSVIMG
olc20_veronii_A LMYGIVMIFNRLS---HGEDSALIRITLFFTAFVFFVP------KTELIKKYAIYGAITG
olc11_veronii_A ILIAVVFAGYHIF---RGEVFSAPRAILVSLLYLLVVP------WHRIQKQMVLLVILFG
olc25_sp_ZOR000 FSVVVFFSLYSIF---RGEQFSMPRTILVSLLYIVIVP------WQMISLNIVRLSIVLG
olc7_veronii_B5 IFYFMVQAISISL---DNGALKELDRPSRALMAAIILPLL---SKHRVHLVSIMSGFALG
olc12_caviae_42 FAYVFVQGLSIWL---DGGKLREFDRPSRVLMAAITLPLL---SRYPVRLVSLIFGVAVG
olc13_media_423 FAYVFVQGLSIWL---DGGKLREFDRPSRVLMAAMTLPLL---SRYPARLVSLIFGFAVG
olc14_media_ws  FAYVFVQGLSIWL---DGGKLREFDRPSRVLMAAMTLPLL---SRYPARLVSLIFGFAVG
olc15_allosacch ILYALVQGLSILL---DNGSVREFDRPSRVIMATLILPLL---VQNPVRPITISLGVAVG
olc9_allosaccha VSFFVIQAVSIGI---DGGTLKELDRPSRLLMAAMTLPLL---SVFPININVLIAGLSIG
olc19_sobria_42 ISFFIIQAVSVWV---DGGVLKELDRPSRLLMAAMTLPLL---SLFPVNINVLLAGLSIG
olc24_salmonici IAFFIIQAVSIWI---DGGTLKELDRPSRLLMAAITLPLL---SALPININVLIAGLSIG
olc23_145       IAFFITQAISIWI---DDGSLKELDRPSRLLMAAMILPLL---SVFPVNINFFIAGLSIG
olc17_jandaei_4 LSYFVLFLISVIF---DGSSFSQLDRPSRALMAALVIPTL---ARHKVKLEVLLCGFACG
olc18_sp_EERV15 LSYFVLFVISVIV---DGSSFSQLDRPSRALMAALVIPTL---AKHKVKLEVLLCGFACG
olc26_encheleia FLYFLVFLVSTML---DSRELSQLDRPSRALMAMLTLPLL---ARHKVKLDVLLFGFGVG
olc8_bv_sobria_ VFYFVVMSISVWL---DGGRISEIDRASRALMAAAMLPLL---ARVPVRLPMLLSGIGIG
olc10_caviae_83 SLYFLVMVISVWF---DGGKISQIDRASRALMAVVILPLL---ACVPVRLPILLSGCGVG
olc28_caviae_42 LLYFLVMSLSVWL---DGGKFSEIDRASKALMAAAILPLV---ACMPVRLNILLSGCGIG
olc3_caviae_A23 VVQSLSWVASRQFVPDYALKYPSVKSMAYLFFFLGLAYWL---KGDPRRIVITLLTFCLG
olc27_simiae    LVQCIPWVIGHFTSLPTAEGNPGLDRLLKLYLFLLGSTLL---EGRERRVFWLWGCAALG
olc16_diversa   IVVFIVGLFSRLL---SPGDIRDVGYFFYKGLFLVVFPILLLTFRDKENRQLAFVLSMAG
olc29_veronii_1 IAVFLFGVFAKII---SPSSFVDIGHFFYKGMFFLVFPALVISLREKTNRQLAFSISMLG
                                                                           *

olc1_piscicola_ GMGL-----GMLSIWQYQ-H-----GMVRVEG-FTNAILFSQAALTLAILN-WF-----V
olc2_salmonicid GIGL-----GIMSLWQYQ-S-----GIVRVEG-FTNAILFSQAALTLAILN-WF-----V
olc22_tecta     GIGL-----GLLSLWQYE-N-----GIVRVEG-FTNAILFSQSALTLVILN-WC-----L
olc4_veronii_Hm GFGL-----GVISIWQYN-H-----GVARVEG-FTNAIIFSQTALTLAILN-FW-----F
olc5_popoffi    GIAI-----GLLAIWQYH-N-----GMLRVEG-FTNAILFAQGALILAILN-SF------
olc6_enteropelo GISI-----GLLAIWQHQ-N-----GMFRVEG-FTNAILFAQGALILVILN-GF------
olc21_taiwanens GISI-----GGLSVWQYY-H-----GLDRVEG-YTNAILFSQAALVMFILN-MM------
olc20_veronii_A GWIV-----GFLAIKEVS-E-----GISRVGG-YTNAILFAQGALVLFILN-LH-----V
olc11_veronii_A GCVA-----GAVGLYEYAVL-----DIRRVGG-VINQIPFALYVAITLLIA-------IY
olc25_sp_ZOR000 GVAA-----GILGVYEYAIL-----DISRIGG-VVNQIPFTLYVAITCVIS-------IA
olc7_veronii_B5 AVIS-----GFIAIYDKIYI-----GMERAFG-DGMPIQSGNISMTLGLFC-LCA---YF
olc12_caviae_42 ACIA-----GSIAIYDKFYL-----GMERAFG-DIMPIQAGNISMTLGLLC-LCG---YF
olc13_media_423 ACIA-----GAIAIYDKFYL-----GMERAFG-DIMPIQAGNISMTLGLLC-LCG---YF
olc14_media_ws  ACIA-----GAIAIYDKFYL-----GMERAFG-DIMPIQAGNISMTLGLLC-LCG---YF
olc15_allosacch AFIA-----GIVSSYEKFYL-----GVDRAFE-NMMPIQGGDISMTLGVLS-LCS---YF
olc9_allosaccha AVSS-----GLIAIYEKFYL-----GMPRAFD-ESMPIQSGNISMTLALLC-LCA---FF
olc19_sobria_42 AISS-----GLIAIYEKVYL-----RMPRAFD-DSMPIQSGNISMTLALLC-LCA---FF
olc24_salmonici AISS-----GLIAIYERFYL-----GMPRAFY-ASMPIQSGNISMTLALLC-LCA---FF
olc23_145       AISS-----GLIAIYEKVYL-----RMPRAFD-DSMPIQSGNISMTLALLC-LCA---FF
olc17_jandaei_4 ALIA-----ACIAIYDKFYL-----GYERAFEFYSIPIQSGNISMSLGLFC-LCG---YF
olc18_sp_EERV15 ALIA-----AGIAIYDKFYL-----GYERAFEFYSMPIQSGNISMSLGLFC-LCG---YF
olc26_encheleia AIIS-----ACIAIYDKFYL-----GYERAFDFYSMPIQTGNISMSLGVFC-LCG---FL
olc8_bv_sobria_ SILA-----FGIAVYDKFVL-----GYERAFS-DMMPIQSGNIAMSLGLFC-LCG---MF
olc10_caviae_83 ALLA-----CGIAIHDKFIL-----GYERAFN-DMMPLQSGNIAMSLGLFC-LCG---LL
olc28_caviae_42 AILA-----FAIAIHDKFFL-----AYERAFG-DVMPIQSGNIAMSLGLFC-LCG---LL
olc3_caviae_A23 VIFTFAYHSSFFSIVETGLD-----GARVDFN-YRNAQHGSLIAGACFLFFVLLL---LT
olc27_simiae    LLAVVL---VQLPEWQRGLK-----GMRVDFG-IRNAQHTAMFFGILLLGC-VCFAGRWL
olc16_diversa   FSIS-----VFMSFIQAFILPDAGWNGERVSG-LWDILRWAEITTFVFVFL-------LA
olc29_veronii_1 FLLS-----VLWSFVQAFVLLPHTWGGERFGG-FWDIGRWAEITTFVFAFM-------LP
                               :
```

```
olc1_piscicola_ FQQRWLPRWFRRGALIGLIAALFALYLSQSRGVWLALGVILAYVIC-YKAYFKPWKYVAV
olc2_salmonicid FQQRQLLRWIRYGALVALVAALFALYLSQSRGGWLALGGIVGYVIC-YKALFKPWKYIAI
olc22_tecta     FIRARKVCWVKTGSLVSITASLLALYLSQSRGVWLALGAVIGLLII-YKSLYKPLKYAFI
olc4_veronii_Hm SVSFSENRLEKWFHIISALFSLGALYLSQSRGVWLAFMVVITCFLL-FKIKTKPLKYISI
olc5_popoffi    FAQESSSLLARCLYIISSIFAISAIYLSQSRGAWLALVVVVTFVLF-VKAIKEPIKYFSW
olc6_enteropelo FGQDESNKFVKCGYLIGAGAAVIAIYLSQSRGVWLALLMLAMLALS-IKATKKPVKYMAL
olc21_taiwanens VVFSCQRIYVSFLASVAVLGSLYALYQSQSRGVWIAAVFIIMIVSF-VKLKHKPIKLSLL
olc20_veronii_A LFHESQAITKKLITIIGVGLSLVAIYLSQSRGVWLSLIIVMVVYFT-VN-RHILIKNIRI
olc11_veronii_A TVLNNQNRNVNLLVWLSIIGSVFAIVMSEVRGVWLALIFTAIILVC-YQLKKWTVRRMAS
olc25_sp_ZOR000 TYLQCDNKYLKVLSVVSILGSVFAIIMTEVRGVWLSIILTIISLVL-FQLKRFGARRISL
olc7_veronii_B5 WHRENNEYVFSKIMLAACLMGMLGSFLSGTRGGWVLLPVIITTIVMHYQRSGRRYFLWVF
olc12_caviae_42 WYKSKGNIKTAMFMLFACAMGMLGSFLSGTRGGWVLLPIIILTIVNHFRGNLCRIDKIVS
olc13_media_423 WYKSKGNVKTAMFMLFACAMGMLGSFLSGTRGGWVLLPLIILTIISHFRGNLCRIDKIVA
olc14_media_ws  WYKSKGNVKTAMFMLFACAMGMLGSFLSGTRGGWVLLPLIILTIISHFRGNLCRIDKIVA
olc15_allosacch WFKKNGSFIIAMFMLASCAMGMLGSFLSGTRGGWLLLPIILFTIVFHFKETLCKVDKMVF
olc9_allosaccha WFRHLGKKSIAAFMLLSFLMGGIGSFLSGTRGGWVLLPIILFTIVFYFRKQLSLKDGLYA
olc19_sobria_42 WFRNIGRKGIAAFMLLSFLMGGIGSFLSGTRGGWVLLPIVLLTIVFYFRKQLSLKDGLYA
olc24_salmonici WFRYRGKKGIAAFLLLSFLMGGIGSFLSGTRGGWVLLPIVLLTIIFYFRKQLCLKDGIYA
olc23_145       WFRSIGRNKISAFMLFSFLMGGMGSFLSGTRGGWVLLPIILVTIVFYFRRKLCLKDGLYA
olc17_jandaei_4 WSRANKFYNYSFFYMIGAISGLIGSFLSGSRGGWILLPVVIFSILMLYRNLIASNERKGL
olc18_sp_EERV15 WYRAKNAKYFSYLYMLGAISGFTGSFLSGARGGWVLLPVVIISILMLYRKIITNNEKKGL
olc26_encheleia WCRAKEKRRRSYFYLLSTLAGLTGSFLSGTRGGWVLLPVVVFSILFFYRGAISKRDRKWL
olc8_bv_sobria_ WAQKKERLAFSLFMLLGACGGMGASFLSGTRGGWVLLPVILLTIGMVFKEYLYRKGSIAL
olc10_caviae_83 WAQKKGKLAFSFFMLLGGCAGMGASFLSGTRGGWVLLPVILFTIAVLFKECLYRKTTLVM
olc28_caviae_42 WAQKKGKLAFALFMLVGTCAGMGASFLSGSRGGWVMLPVILITIAMLFKECLYRKGTLIM
olc3_caviae_A23 SKFRKLSLGNAGILLGILLFGVFSVIL-QSRQSWLAISASLAILPLLGGGEHWPRRKVVL
olc27_simiae    SARRRQWLWRLPLLLAVLAVAVLGIVVTQTRAVFLAVGAAAILGVVLWGLMQRPHWKNLL
olc16_diversa   KLFDENALHKKYVIFLCMCFVAVSLILSGGRAGWVAVIVTSTLYML-FLNKKYLIVSALL
olc29_veronii_1 KLSDDMTASKKVALGLFLFVTFLSLIISGGRAGWIAASFSFVVYMI-FMNRKMLYVFTPV
                                       .      *  ::

olc1_piscicola_ AI-LCT--ATTGAIY-HTNQLVQVRVAEA---VSDINS-AEKGSYD----SSWGLRVIAW
olc2_salmonicid AM-LCI--ASIGITY-HTNQLVQLRVADA---VSDLNF-AEKGSYN----SSWGLRVVAW
olc22_tecta     TL-LLV--ALAGGLY-QTNTIVQGRMAEA---VSDIKG-IEGGTYY----SSWGLRVVAW
olc4_veronii_Hm TL-GLL--LVLGMLC-LNSSIVKNRIDSG---LSDVVN-MENGNYS----TSLGLRAVAW
olc5_popoffi    FL-ILS--CVLVITF-YESDMLRARILES---ISEVNM-MESNDYT----SSWGLRLMAW
olc6_enteropelo FL-IVF--ILLTIVF-SETEVFKIRIQES---LSELSM-MESNNYN----TSWGLRVMAW
olc21_taiwanens IL-AFI--ISMILLY-QYSPVVQQRILDA---TSDLDK-MQGNSYD----TSWGLRLMAW
olc20_veronii_A IL-PGL--IIILVAFNYNSNIFIQRMDAV---KNNFIQ-MDKGNYQ----TSIGLRFVVW
olc11_veronii_A MA-VVA--LAMLVLL-SLIPAIEQRVDET---RQEITQ-ISAGNKD----TSIGIRLQLW
olc25_sp_ZOR000 IT-AAT--LIVLFSF-YTIPELSQRVNVT---KQEFVQ-ISKGNMD----TSIGLRFQLW
olc7_veronii_B5 TC-IVI--GGFGIIS-TPQTGVLDRIKTA---KSDIVQFIDGENPD----TSLGIRFQLW
olc12_caviae_42 AV-FGL--GLLAIVA-IPQTGVAERIHVA---RSDIVQFMDGGNKN----TSIGIRFQLW
olc13_media_423 AV-FGL--GLLAVVA-IPQTGVAERIQVA---RSDIVQFVDGGNKN----TSIGIRFQLW
olc14_media_ws  AV-FGL--GLLAVVA-IPQTGVAERIQVA---RSDIVQFVDGGNKN----TSIGIRFQLW
olc15_allosacch AI-LML--GMIGVVS-IPQTNVLVRIDAA---RSDVVHFIKGDNKD----TSLGIRFQLW
olc9_allosaccha ST-VCA--LLLFLAM-LPQSGVMDRIYNA---KADVVHYFDGVNPD----TSLGIRLQLW
olc19_sobria_42 ST-VCA--LLIFLAM-LPQSGVMDRISNA---KADVVHYFDGVNPD----TSLGIRLQLW
olc24_salmonici ST-VCA--LLLFLAT-LPQSGVMDRIYTA---KEDVVHYLDGVNPD----TSLGIRLQLW
olc23_145       GS-VCV--LLLFLAL-LPQSGVMDRIYAA---KEDIVHYIDGLNPD----TSLGIRLQLW
olc17_jandaei_4 LC-LLF--IIIAMVL-APQTGMLKRFDAA---KTDIEQYVDGTNLD----TSLGIRFQLW
olc18_sp_EERV15 LF-LFA--IVIAMVL-TPQTGMLKRFDAA---KTDIEQYIDGTNLD----TSLGIRFQLW
olc26_encheleia LL-ILV--TVSSIVF-TPQSGVIKRVYEA---HSDIVRYIDGSERN----TSLGIRFQLW
olc8_bv_sobria_ ITCILL--CGGILVL-QPQSGVEARIEQA---QHDISQYLDKTNLN----TSLGIRLQLW
olc10_caviae_83 LVGGLL--CGGFLIV-QPQSGVEARIELA---QNDISQYLDKTNLN----TSLGIRLQLW
olc28_caviae_42 VISVLL--CGVFFVV-QPESGVEKRIELA---QRDISQYMDKTNLN----TSLGIRLQLW
olc3_caviae_A23 TY-AIL--LMTMLAL-YQVSFIRERLLAGFVHQGDVHA-ILTGNWNEVRDFSIGVRLKTW
olc27_simiae    LV-LALGGVLIGTTLHYFKSQTIGRSGAE---ANVIEM-VWEGKLDRIPYTSVGTRVNTW
olc16_diversa   LG-AGT--FIIGKWEPEKIEFVMGRALSV----------TE-TKED----YSNYSRLLMW
olc29_veronii_1 AC-VLI--FSLYLAMPSQFNAVAGRATSV----------TETTSKD----YSNFSRILMW
                                        *                          *   *   *
```

```
olc1_piscicola_ QSAWLGFLD-------SPVAGVGTDGFDALKQTQINNHQVSPLIF-----HPALVHSHNQ
olc2_salmonicid QSAWLGFLD-------APLTGVGTNGFDALKQEQVARGLVPPLAL-----NAALAHAHSQ
olc22_tecta     ESAWLGFLE-------SPLIGVGTDGFTAVKEQQISQGLVSPLVM-----DPALAHAHSQ
olc4_veronii_Hm ESAWLGFLD-------SPLIGVGTEGFDALKQEQVKLGLVPQALL-----NPELAHAHNQ
olc5_popoffi    RSAWAGFVD-------NPLLGVGLDGFKTMQQQQLLDGIVNKFYV-----DYGMYHAHNQ
olc6_enteropelo KSAWFGFIE-------HPLLGVGLDGFKNIQHQQLLDGIVDNFYV-----NYGMYHAHNQ
olc21_taiwanens KSAWFGFLE-------YPIFGIGYHGLDGLRLDQLKLGIVDQFYI-----NNGVYHAHNQ
olc20_veronii_A KSAWLGFLE-------YPFIGVGKDGIDELKKQQVSEHKINPVLLV-GDAGLGMPHAHNQ
olc11_veronii_A HSAIEIIKA-------HPLMGVGTKGYQNIMEQQYQQGVITSAVL-----SFKNAHYHNQ
olc25_sp_ZOR000 HSAIEIIKD-------HPLMGVGTKKYPDLMVRQYQQGMISELAL-----SFKDAHFHNQ
olc7_veronii_B5 SSAWDAFQK-------KPIFGWGNNGIRQIHKQQLEQGKISEFIY-----NFN-YHAHNQ
olc12_caviae_42 YSAFDAFQK-------KPLFGWGNNGLQQAHTVQLAAGEISEFLY-----KFN-FHAHNQ
olc13_media_423 YSALDAFQK-------KPLFGWGNNGLQQAHAAQLAAGEISDFLY-----DFN-FHAHNQ
olc14_media_ws  YSALDAFQK-------KPLFGWGNNGLQQAHAAQLAAGEISDFLY-----DFN-FHAHNQ
olc15_allosacch YSALDSYLH-------KPFIGWGNNGIRQSQLEQLKSGKITQFIY-----DFD-SHAHNQ
olc9_allosaccha ESAYNSFLE-------KPIFGWGNNGIRLSQLNQYNNGSISEFIY-----NFN-SHAHNQ
olc19_sobria_42 ESAYNSFLE-------KPIFGWGNNGIRLSQLNQYNNGSISEFIY-----NFN-SHAHNQ
olc24_salmonici ESAYNSFLE-------KPIFGWGNNGVRLSQLNQLNNGAMSEFIY-----NFN-SHAHNQ
olc23_145       DSAYHSFLE-------KPIFGWGNNGVRLSQLNQYNNGSISEFIY-----NFN-SHAHNQ
olc17_jandaei_4 RSAWDSFKE-------KPFFGWGQDGLRESQKQQLKENKITNFIY-----NFN-YHAHNQ
olc18_sp_EERV15 GSAWDSFKE-------RPFFGWGQDGLRESQKQQLQKNEITNFIY-----NFN-YHAHNQ
olc26_encheleia RSAWDSFIE-------KPILGWGHDGLRISQKQQLDAGKITEFIY-----NFN-YHAHNQ
olc8_bv_sobria_ QSAWQSFTQ-------KPLFGWGNHGIRISQQEQLGQGEISQFIY-----DFN-YHAHNQ
olc10_caviae_83 QSAWQSFTE-------KPLFGWGNHGIRQSQKAQLARGEISQFIY-----DFN-SHAHNQ
olc28_caviae_42 KSAWQSFTE-------KPLFGWGNHGIRESQKAQLEQGKITQFIF-----NFN-SHAHNQ
olc3_caviae_A23 LEAIQWFLA-------HPFFGTGYGS----QSLVITLSKTLPDYI-----TREFRHLHNS
olc27_simiae    LVAWEKIKE-------RPLFGWGREA----RSWVIEQSPTLPAWV-----KQEFGHLHNY
olc16_diversa   GNGLKWLEANMVDNPRQFMFGIGFKRFEKEYTEYLDSISDSRELINITHGNYSMRDLHNA
olc29_veronii_1 GNGVSLLHENLSHYPRKFLFGIGFNKFQTEYSAYLNKISNTEDLIKLTQGNYSLNDLHNS
                  .               . * *                                . *.

olc1_piscicola_ YMQNLVVRGGIGFVALVIFLGLPL------YFAAKNINRI----------SAGVLVPLAF
olc2_salmonicid YMQNLVIRGGIGFVVLVAFLFLPL------WLSMKKMGGD----------AACVLIPLSF
olc22_tecta     YMQNLIIRGAVGILALAIFLFYPM------KLLVERAGWG----------SPYTMIPLSF
olc4_veronii_Hm YLQTLVIRGGIGFIFLLGILILPA------IFFAKEMGFV----------SAGVFIPISF
olc5_popoffi    FIQNMVIRGILGLLAALAILVYPM------WLFWQQSKLV----------SVGFIITLST
olc6_enteropelo FMQNMVVRGILGLLVAIAVLVYPM------IFFYHRTKVL----------SAGFMVSFGV
olc21_taiwanens FMQNILIRGMLGGVVAILVLIYPI------VIARRSFGVF----------SASTLVPIGI
olc20_veronii_A FLNQLVMRGLVGFIPT--------------------------------------------
olc11_veronii_A YLDSYVVRYGVPGLLIVFVIFMSPY------LLYGLQCTPQ---------GFLCISISSVC
olc25_sp_ZOR000 YLDSYVVRYGLIGVLAIIAIFLSPL------FLYGKLDVGV---------KNICLAIALIC
olc7_veronii_B5 FLDEMAKRGIIGLFALCLVYFYPI------SVFYKYKKSLSMKY------ILMAVASLTL
olc12_caviae_42 FLDEMAKRGLIGLLVLLIMYLYPL------YLFYKHRRGDSLLP------IMLAVTSLTL
olc13_media_423 FFDEMAKRGVIGLLALLSMYLYPL------YLFYKHRGDDSLFP------IMLAVTSLTL
olc14_media_ws  FFDEMAKRGVIGLLALLSMYLYPL------YLFYKHRGDDSLFP------IMLAVTSLTL
olc15_allosacch FLDEMSKRGTLGLVVILMMFLYPV------LIYRRLTKINLITP------IMLGVSVLCT
olc9_allosaccha FLDELAKRGIIGFLMLALLYFYPL------WVASKNRNNLLVS-------SMLAVSSLTL
olc19_sobria_42 FLDELAKRGAVGFCMLVMLYLYPL------WVASKNKNNLLVS-------SMLAVSSLTL
olc24_salmonici FLDELAKRGAVGFLMLVMLYFYPL------WVASKNKNNLLVS-------SMLAVSSLTL
olc23_145       FLDELAKRGIIGLLMLTLLYFYPL------WVASRNRDNLLVS-------SMLAVSSLTL
olc17_jandaei_4 YLEEMAKRGIIGLLVLIAMLCVPI------ILVKKRFSIMAQESLAYSGGVLVIVTCLTT
olc18_sp_EERV15 YLEEMAKRGVMGLFVLLAMLSLPV------VLVKSKMSCMREQSAAYGASVIVIVTCLTT
olc26_encheleia YLEDMAKRGLIGLSSLLGILFIPM------VIAKSRMVGASITSSVFGGLTIVMATCLTT
olc8_bv_sobria_ FLDEMAKRGIIGLVAFLLILLTPL------FLVKRRLRAPHDID-VHCGAALVIVTVFSC
olc10_caviae_83 FLDEMAKRGIVGLGALLLMMLIPL------FLVKSRLHRPHNDD-VHCGSALVIVAVFSS
olc28_caviae_42 FLDEMAKRGILGLGSLLFMLIAPV------LIVRRKLRQPHNID-VHCGATLLIVTTFSS
olc3_caviae_A23 NMETLVCWGVAGFLVLYGTLFSVLRQVLCCPVSSRF----------------IKMLAISF
olc27_simiae    FIEIQLSYGLAGSLWLLGMVVATL---WGCWAAWRRGDMP----------LDVCLFGFCF
olc16_diversa   YIDSSNKMGVLYTVLFYAFLVFVA------YSFIRNGIAH----------QGVGLILSFV
olc29_veronii_1 YLDSANKMGFLYTLFFYGSLIFIV------YLFYKKESLI----------KGCILIAPFL
                 ::     *
```

```
olc1_piscicola_ AINSL----SDVPFEHQGVLYLYTLSLVFIWFAHE--------FEKDTCTS--------
olc2_salmonicid AISAF----SDVPFEHQDILYLYVLSMIFIWYSSE--------LRENKSAL--------
olc22_tecta     AVSAL----SDVPFEHQNTLYLYALSLVFCWCAIE--------FNMEKKAS--------
olc4_veronii_Hm AVGAV----FDVPFEHQSMMYLYSFSLIFILFCHE--------LKQD-------L---L
olc5_popoffi    FVCAL----TDVPIEHQNTLYIYLISLFFIFFKNE--------GRFNEKVVL-------
olc6_enteropelo FVCAL----SDVPMEHQNTLYIYMITLFSLCLSYE--------IEKNKEAMS-------
olc21_taiwanens LICSL----SDVPLEHQNTLYIYTLSLVLCWFYYENAH-LLEGVNSEEGISEK------
olc20_veronii_A -----------------------------------------------------------
olc11_veronii_A LIAGL----TDVPLIHTGIIYVIILYSAVIYLTSC--------DYKKTQL---------
olc25_sp_ZOR000 IFAAL----TDVPLIHTGIIYILIIYPAAIFLSCS--------NNASGEVLNY-----T
olc7_veronii_B5 IDYSL----SQAFISHNSGIVFFFVCFSLLITSLN--------ETNKEEV---------
olc12_caviae_42 IDYSL----SQAFISHNSGITFVSFLFLVEITMLN--------NSAEIRKNNE-----K
olc13_media_423 VDYSL----SQAFISHNSGITFVSFLLLVKITMLN--------NSAEIRKNNA-----E
olc14_media_ws  VDYSL----SQAFISHNSGITFVSFLLLVKITMLN--------NSAEIRKNNA-----E
olc15_allosacch IDYSL----SQAFLNHNSGIIFFSMLTAILVTSAV--------SEQMMMDR--------
olc9_allosaccha VDYSL----SQAFISHNSGMIFFMFLFSALITTIK--------VSPLEGRK--------
olc19_sobria_42 IDYSL----SQAFINHNSGIIFFMFLFSVLISMFS--------TNSTSEALND------
olc24_salmonici IDYSL----SQAFINHNSGIIFFMFLFSVLITALP--------TVPTNSKEINVEAKSD
olc23_145       VDYSL----SQAFINHNSGMIFFMFLFSALITTIK--------ASPLEERK--------
olc17_jandaei_4 ADYHL----SQAYFSHNSGISFFVFSLVLALSVSM--------DDGNH-----------
olc18_sp_EERV15 ADYHI----SQSYFAHNSGITFFIFSMVFGFSATF--------KDAEVLL---------
olc26_encheleia IDYNL----SQAYFAHNSGITFFIFSLVLSLSASL--------EGKTQS----------
olc8_bv_sobria_ IDYSL----SQAFLGHNSGSTFVFSSLVIFMSIVF--------GGPNGIK---------
olc10_caviae_83 IDYCL----SQAFFGHNSGITFFVSSLVIAASIVF--------KESDTE----------
olc28_caviae_42 IDYCL----SQAFFGHNSGISFFVASLVMTASIVF--------GSQNEDSRH-------
olc3_caviae_A23 ILYWLIINNFESFFYMRSGQWVFSVFMGAIYSTALHWELNRLGKEGHENLRH-------
olc27_simiae    VFYFLIVNNFESYFSFWTGGLAFNLIMAGLVSLAWR---RSFPVNAHQ-----------
olc16_diversa   ILGVF----YANYLEFQTSVFFFLLALSHSSV--------AMRRRD-------------
olc29_veronii_1 ILGVF----YTNYLEFQTYIFFFVVALAYSEV--------EQYNVDNHKVRA-------
```

Supplementary Figure 1(**b**). Predicted transmembrane regions plots for O-Antigen ligase domain-containing predicted proteins for each of the types assigned for *wa* Region 1

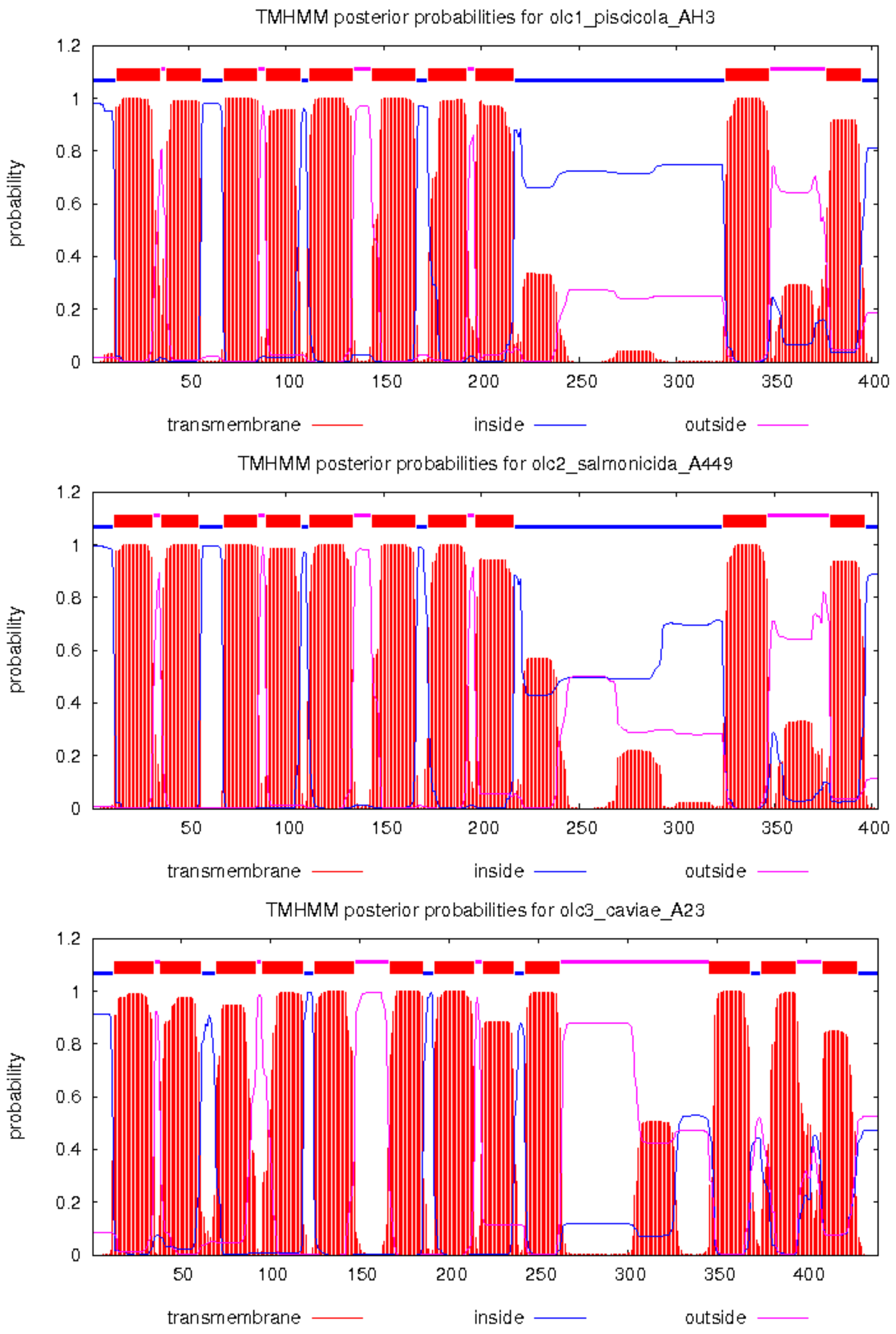

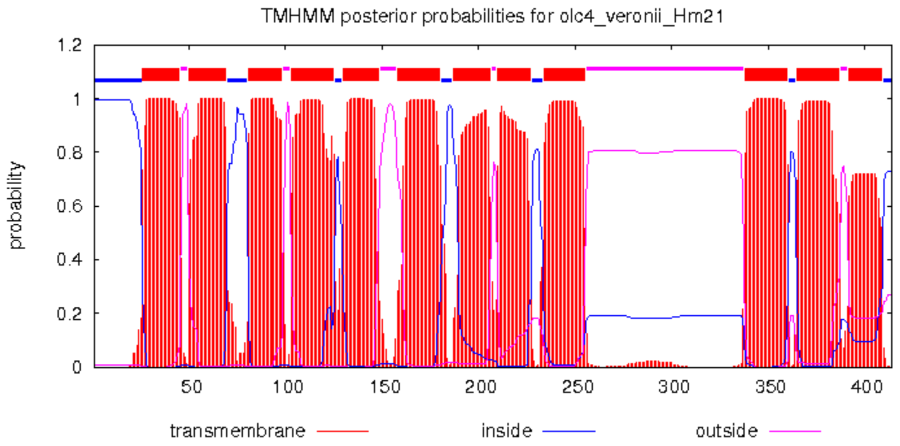
TMHMM posterior probabilities for olc4_veronii_Hm21
probability
1.2
1
0.8
0.6
0.4
0.2
0
50
100
150
200
250
300
350
400
transmembrane
inside
outside

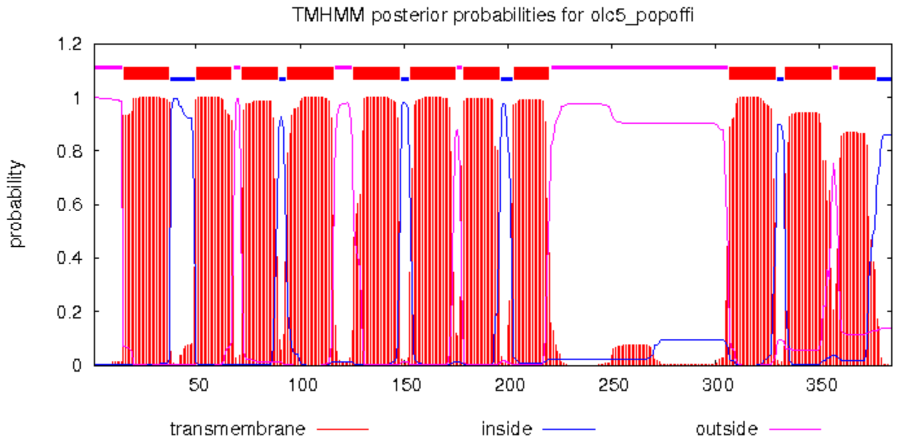
TMHMM posterior probabilities for olc5_popoffi
probability
1.2
1
0.8
0.6
0.4
0.2
0
50
100
150
200
250
300
350
transmembrane
inside
outside

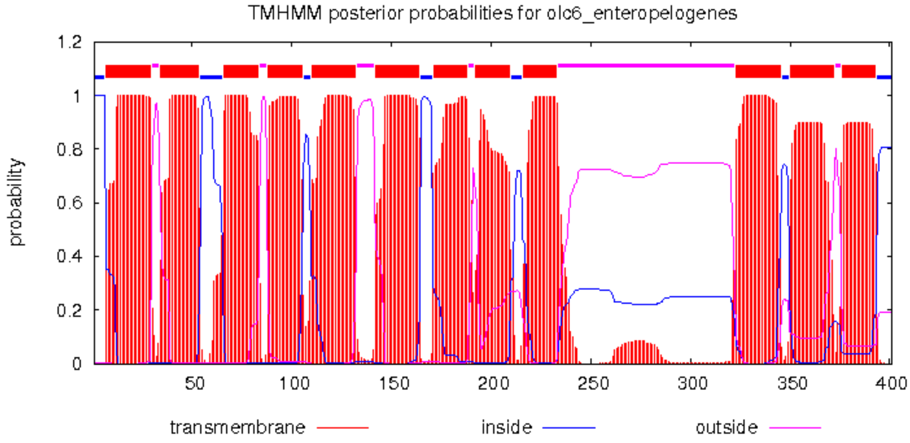
TMHMM posterior probabilities for olc6_enteropelogenes
probability
1.2
1
0.8
0.6
0.4
0.2
0
50
100
150
200
250
300
350
400
transmembrane
inside
outside

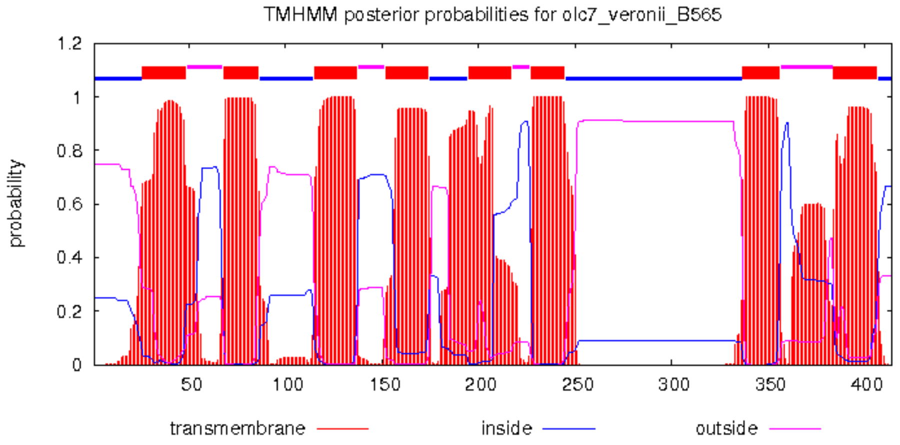
TMHMM posterior probabilities for olc7_veronii_B565
probability
1.2
1
0.8
0.6
0.4
0.2
0
50
100
150
200
250
300
350
400
transmembrane
inside
outside

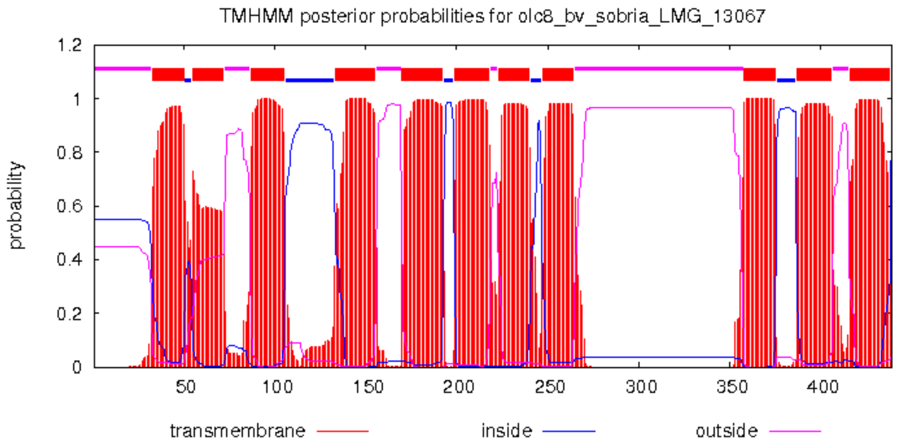
TMHMM posterior probabilities for olc8_bv_sobria_LMG_13067
probability
1.2
1
0.8
0.6
0.4
0.2
0
50
100
150
200
250
300
350
400
transmembrane
inside
outside

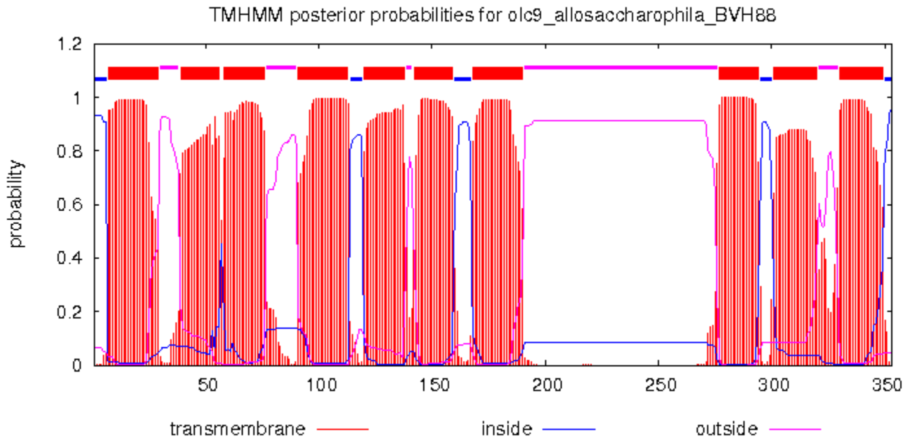
TMHMM posterior probabilities for olc9_allosaccharophila_BVH88
probability
1.2
1
0.8
0.6
0.4
0.2
0
50
100
150
200
250
300
350
transmembrane
inside
outside

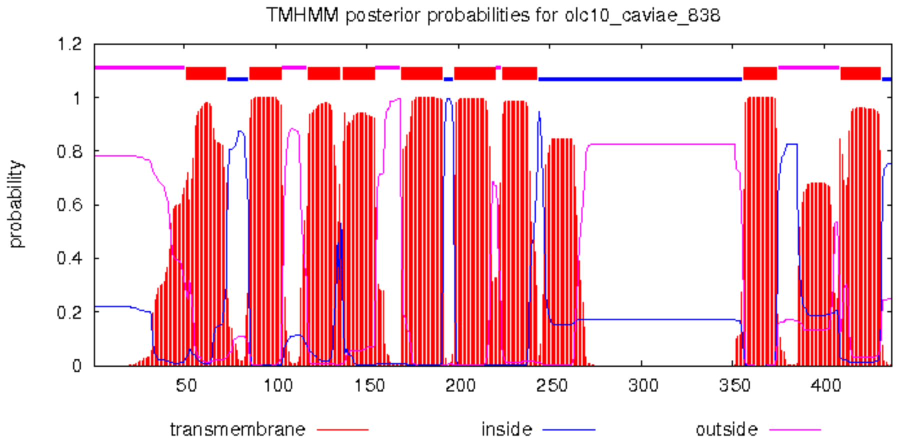
TMHMM posterior probabilities for olc10_caviae_838
probability
1.2
1
0.8
0.6
0.4
0.2
0
50
100
150
200
250
300
350
400
transmembrane
inside
outside

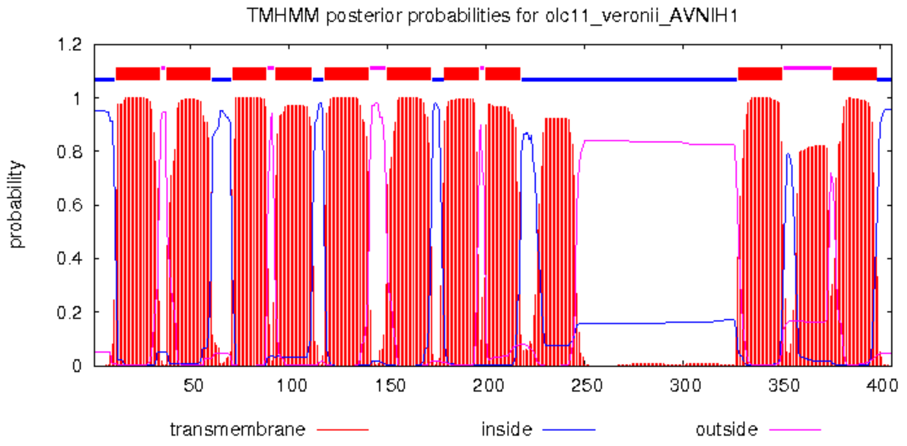
TMHMM posterior probabilities for olc11_veronii_AVNIH1
probability
1.2
1
0.8
0.6
0.4
0.2
0
50
100
150
200
250
300
350
400
transmembrane
inside
outside

TMHMM posterior probabilities for olc12_caviae_4221

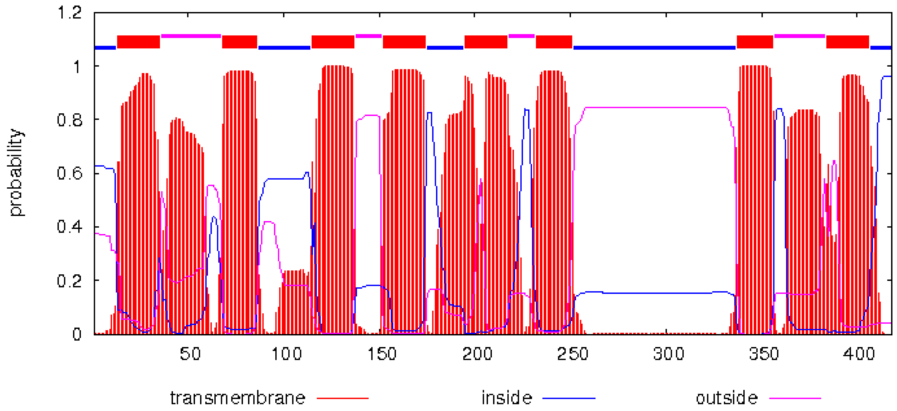

TMHMM posterior probabilities for olc13_media_4232

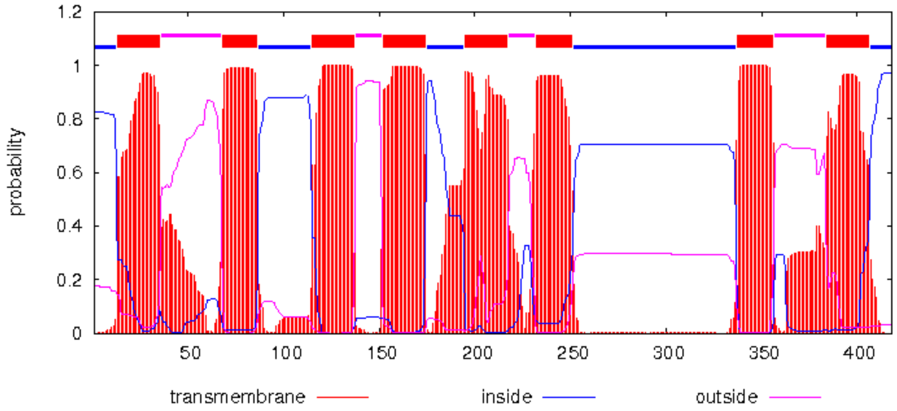

TMHMM posterior probabilities for olc14_media_ws

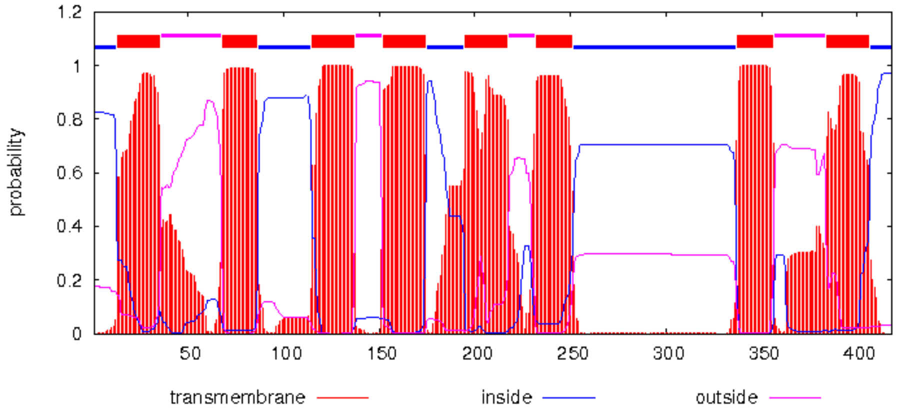

TMHMM posterior probabilities for olc15_allosaccharophila_4199

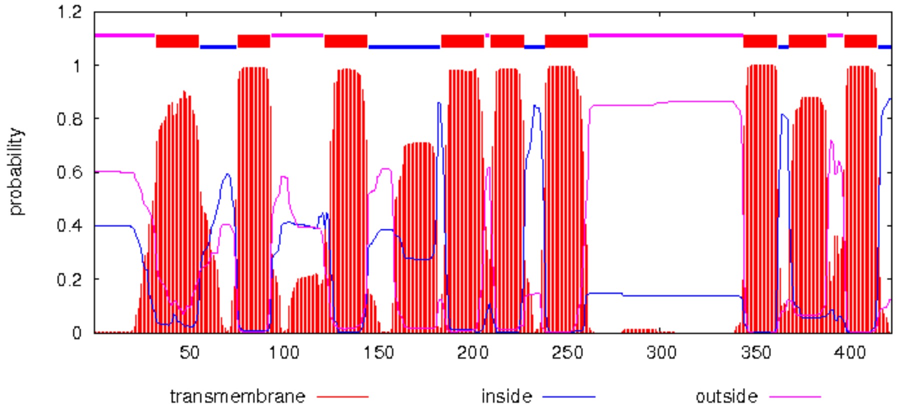

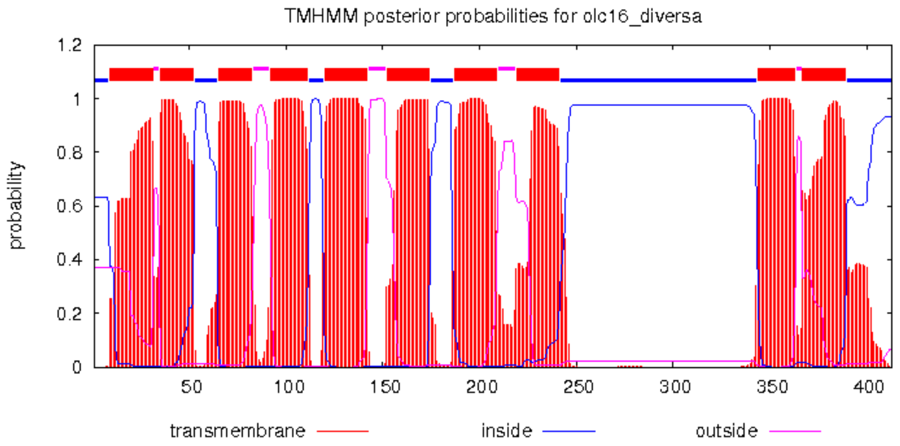
TMHMM posterior probabilities for olc16_diversa
probability
transmembrane
inside
outside

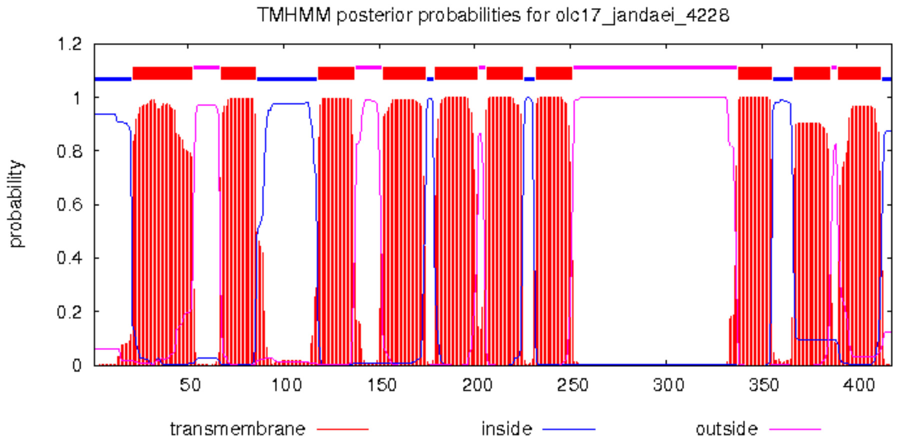
TMHMM posterior probabilities for olc17_jandaei_4228
probability
transmembrane
inside
outside

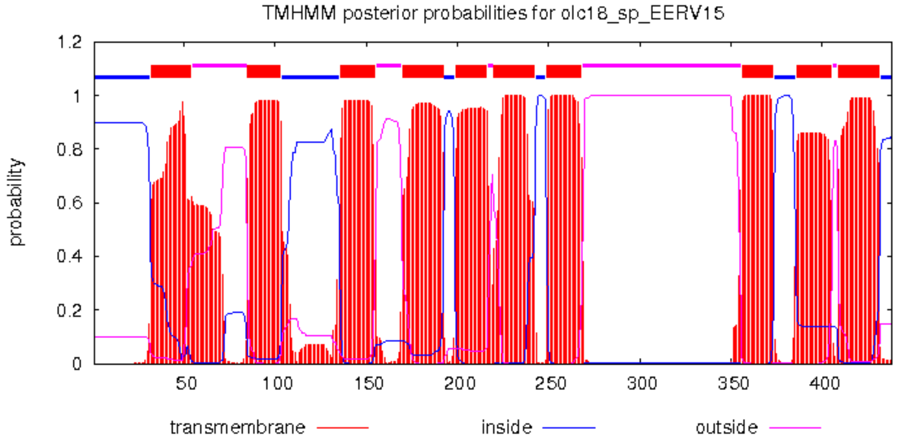
TMHMM posterior probabilities for olc18_sp_EERV15
probability
transmembrane
inside
outside

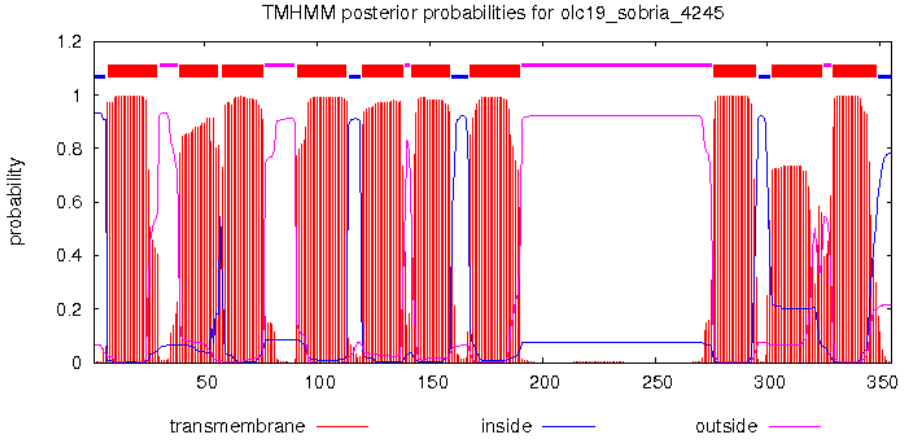
TMHMM posterior probabilities for olc19_sobria_4245
probability
transmembrane
inside
outside

TMHMM posterior probabilities for olc20_veronii_AMC34

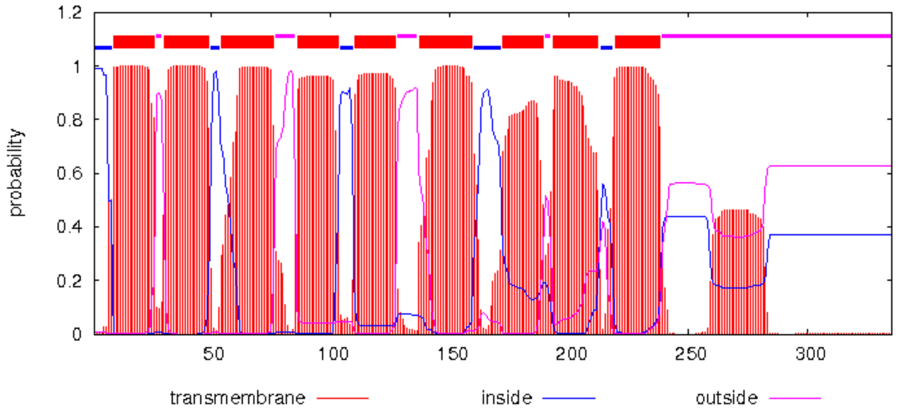

TMHMM posterior probabilities for olc21_taiwanensis

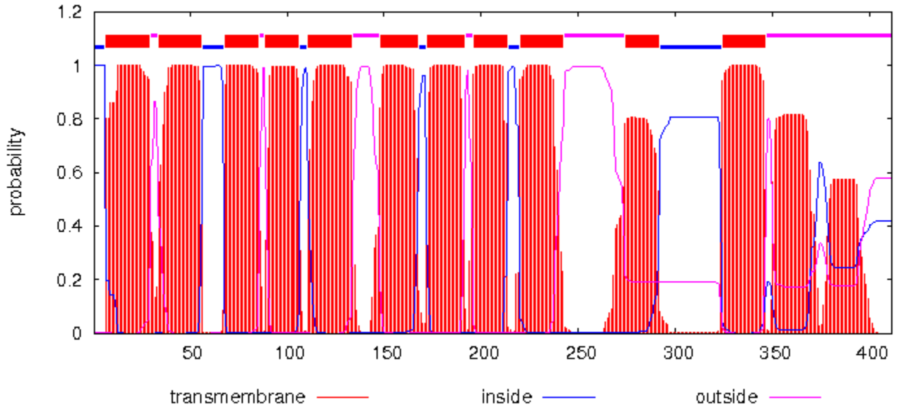

TMHMM posterior probabilities for olc22_tecta

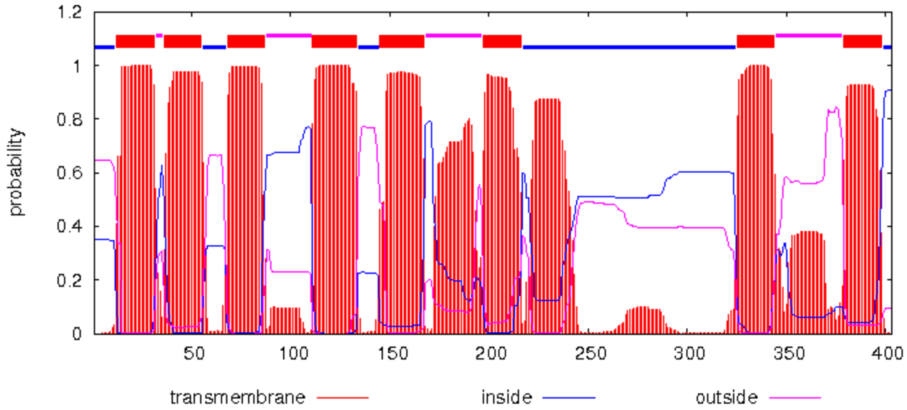

TMHMM posterior probabilities for olc23_145

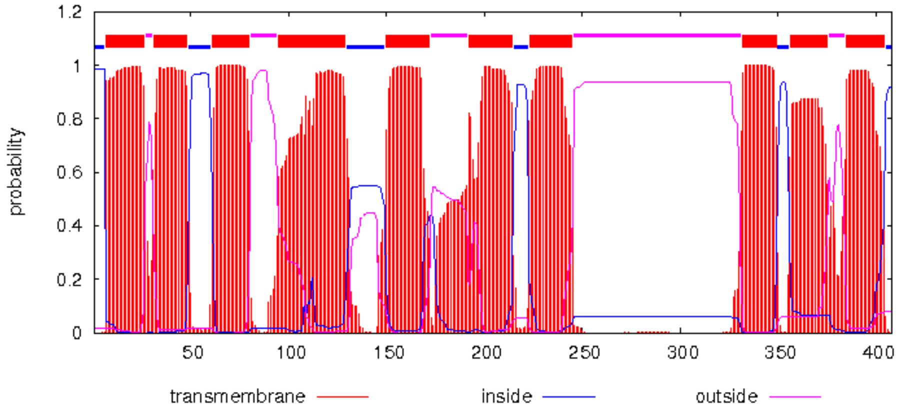

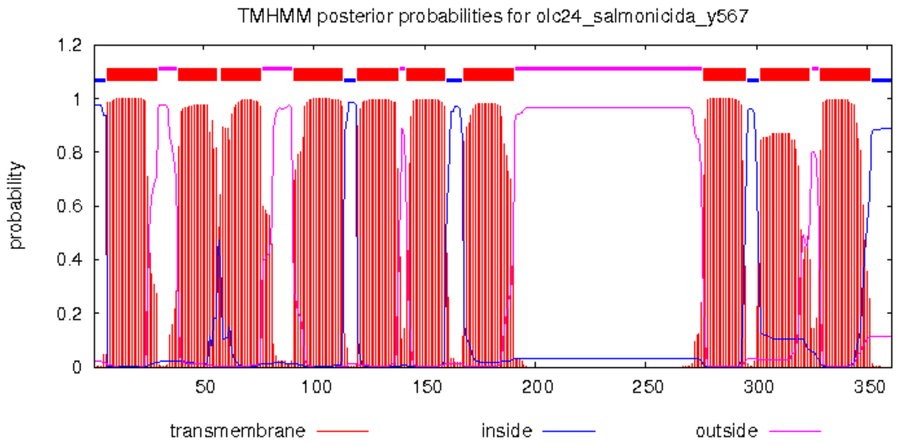
TMHMM posterior probabilities for olc24_salmonicida_y567
probability
1.2
1
0.8
0.6
0.4
0.2
0
50
100
150
200
250
300
350
transmembrane
inside
outside

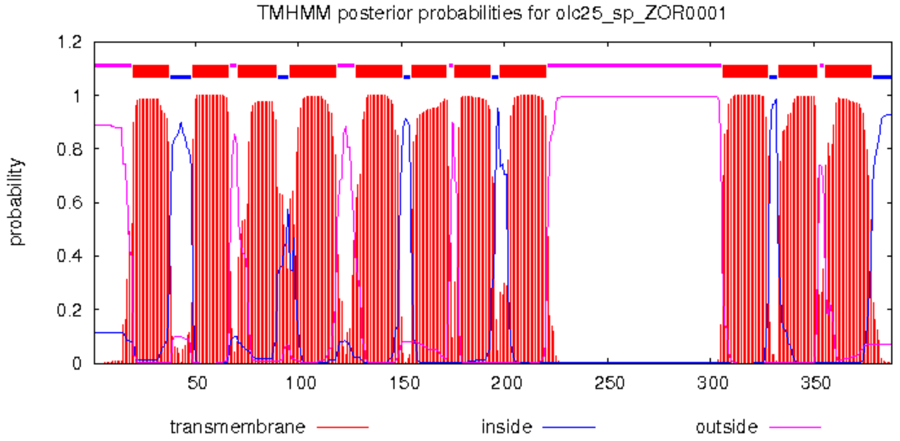
TMHMM posterior probabilities for olc25_sp_ZOR0001
probability
1.2
1
0.8
0.6
0.4
0.2
0
50
100
150
200
250
300
350
transmembrane
inside
outside

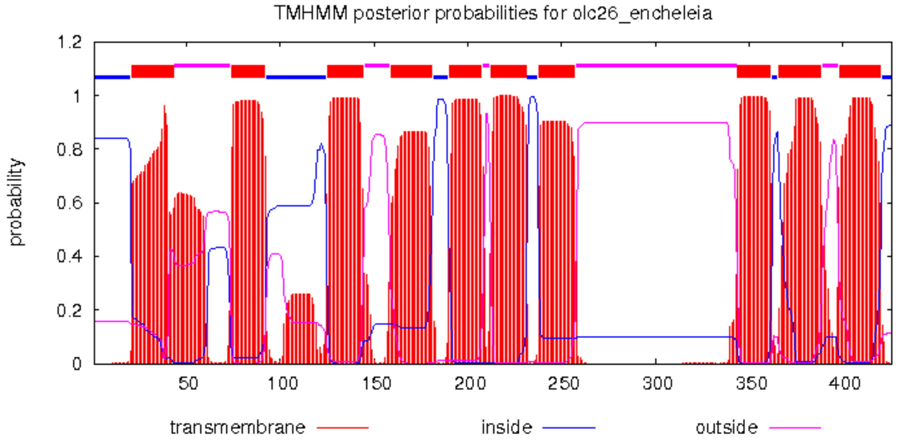
TMHMM posterior probabilities for olc26_encheleia
probability
1.2
1
0.8
0.6
0.4
0.2
0
50
100
150
200
250
300
350
400
transmembrane
inside
outside

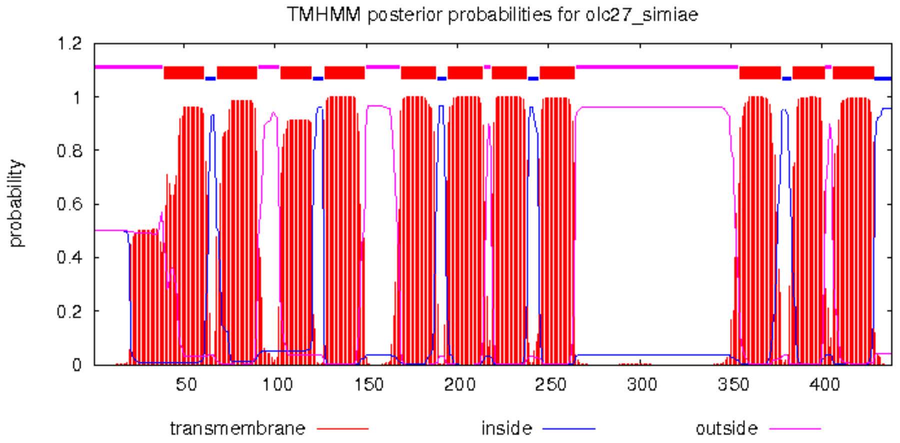
TMHMM posterior probabilities for olc27_simiae
probability
1.2
1
0.8
0.6
0.4
0.2
0
50
100
150
200
250
300
350
400
transmembrane
inside
outside

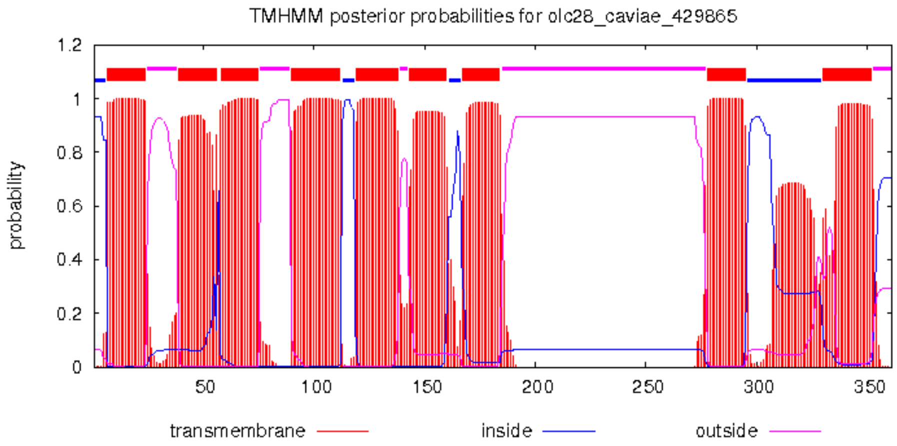
TMHMM posterior probabilities for olc28_caviae_429865
probability
transmembrane
inside
outside

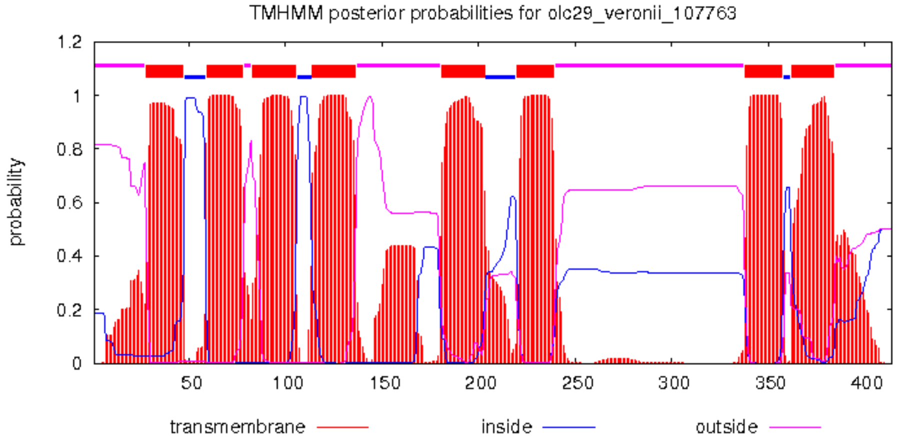
TMHMM posterior probabilities for olc29_veronii_107763
probability
transmembrane
inside
outside
